# Validity, Reliability, Accessibility, and Applicability of Young Children’s Developmental Screening and Assessment Tools across Different Demographics: A Realist Review

**DOI:** 10.3390/children11060745

**Published:** 2024-06-18

**Authors:** Stefan Kurbatfinski, Jelena Komanchuk, Aliyah Dosani, Nicole Letourneau

**Affiliations:** 1 Department of Community Health Sciences, Cumming School of Medicine, University of Calgary, Calgary, AB T2N 1N4, Canada; stefan.kurbatfinski@ucalgary.ca; 2 Faculty of Health and Social Development, School of Nursing, University of British Columbia Okanagan, Kelowna, BC V1V 1V7, Canada; jelena.komanchuk@ubc.ca; 3 Faculty of Health, Community and Education, School of Nursing and Midwifery, Mount Royal University, Calgary, AB T3E 6K6, Canada; adosani@mtroyal.ca; 4 Faculties of Nursing & Cumming School of Medicine, University of Calgary, Calgary, AB T2N 1N4, Canada

**Keywords:** child development, multidomain development tools, screening, assessment, validity and reliability

## Abstract

Valid and reliable developmental screening and assessment tools allow professionals to identify disabilities/delays in children, enabling timely intervention to limit adverse lifelong impacts on health. However, differences in child development related to culture, genetics, and perinatal outcomes may impact tool applicability. This study evaluated the validity, reliability, and accessibility of multidomain developmental screening tools for young children, analyzed the applicability of tools across different contexts, and created a compendium of tools. Employing adapted realist review methods, we searched APA PsycInfo, MEDLINE, CINAHL, ERIC, and Google to identify relevant articles and information. We assessed accessibility, validity, reliability, and contextual applicability (N = 4110 evidence sources) to create tool ratings and make recommendations. Of 33 identified tools, 22 were screening and 11 were assessment tools. Fewer screening tools than assessment tools were rated highly overall. Evidence for use in different cultures was often lacking for both types of tools. The ASQ (screening) and BDI (assessment) tools were rated most favorably and are recommended for use, though other tools may be more applicable in different contexts (e.g., NEPSY among children with Asperger’s Syndrome). Future research should focus on assessing the validity and reliability of tools across different demographics to increase accessibility and ensure all children are properly supported.

## 1. Introduction

Child development is shaped significantly by caregiving environments [1,2], genetics [3], determinants of health [4], and other health outcomes (e.g., asthma; [5]), interacting concomitantly to exert positive or negative impacts. Researchers and health professionals specializing in child development often subdivide child development into four main domains: physical (gross and fine motor), cognitive, social–emotional, and language (Table 1; [6]). Delays in certain domains can lead to health sequelae, such as behavioral concerns, that may persist throughout the lifetime without intervention [7]. Early screening or assessment of children’s developmental trajectories before school age permits the identification of children’s diverse developmental needs, timely intervention if warranted, and prevention of poorer quality of health across the lifespan [8]. Therefore, valid (tools that accurately measure what they are designed to measure) and reliable (tools that reproduce similar results when used again) means of screening and assessing young children’s (newborns to five-year-olds) development are critical to ensuring that early intervention and resources are provided to caregivers in a timely manner to promote healthier development over the lifespan [7,9]. However, ascertainment of the validity and reliability of child development tools across various caregiving, genetic, and perinatal determinants of health is required to increase applicability and accessibility and ensure child development is being accurately screened and assessed.

### 1.1. Caregiving Environments, Genetics, and Child Development

Early environments significantly impact child development, affecting how children behave, respond, and communicate in school, recreational, and other settings [1,2]. Children residing in secure and safe households with caregivers that engage in developmentally appropriate interactions are more likely to acquire the stimulation they need for optimal development and longevity [3,10]. Conversely, children who experience adverse childhood experiences (e.g., physical abuse) are at risk for developmental challenges [2,3]. Studies support the positive association between adverse childhood experiences and worsened child developmental outcomes, which can manifest as behavioral challenges, issues with learning and conduct, social and communicative challenges, attachment insecurity, worsened motor movements, and other mental or physical health problems [11,12]. Tools that are sensitive to childhood adversity can help healthcare professionals identify potentially injurious interactions occurring within the household and help predict future developmental milestones, allowing for the promotion of healthier familial functioning and better monitoring of children’s development.

Children may also inherit genetic factors that promote or challenge their development trajectories (e.g., global developmental delay; [3]). For example, abnormalities in certain neurotransmitters or chromosomes that affect chemical signaling and genetic expression can disrupt children’s appropriate physiological responses to environmental stimuli (e.g., caregiving; [3,13]). Further, certain genotypes may act advantageously in certain conditions while serving disadvantageously in others, reflecting the differential susceptibility phenomenon [14]. On the other hand, children who inherit an irregular number of chromosomes are at risk of developing certain health conditions (e.g., Down syndrome), altering how the developmental trajectory is expected to progress [15]. If healthcare professionals are aware that children have certain genotypes or different chromosomal numbers than expected, they can apply valid and reliable child development tools when available to monitor and predict development and provide adequate support [16]. Overall, the interplay between nurture (caregiving environment) and nature (genetics) can result in positive (e.g., secure attachment) or negative (e.g., insecure attachment) developmental outcomes through epigenetic mechanisms [17,18,19]. High-quality screening and assessment tools, characterized by reliable and valid psychometric properties, are essential to ensure children’s development is being measured accurately in the context of various genotypes and epigenetic expressions.

### 1.2. Perinatal Health Factors

Perinatal factors such as birthweight, gestational age, and the childbirth process can significantly impact children’s development [20,21,22]. Low birthweight in children and preterm births are associated with complications in earlier years with attenuated rates of growth [20,21], resulting in delayed attainment of developmental milestones and challenges with behavior [22]. Complications during childbirth can also impact children’s development. For example, some studies have reported increased externalizing behaviors (e.g., hyperactivity) among 11-year-old children born with birth complications such as breech birth [23] or increased odds of behavioral problems among children born via cesarean section [24]. Another study team suggested similar outcomes among infants, even in cases of mildly stressful birth deliveries [25]. Comprehensive screening and assessment tools must also consider how children’s developmental trajectories may differ based on their perinatal outcomes and be sensitive to such differences. If tools are predictive of how child development is expected to progress in the context of these various perinatal outcomes, healthcare professionals can better intervene and attempt to minimize or even prevent the impacts on developmental outcomes.

### 1.3. Consideration of the Determinants of Health in Young Children’s Developmental Tools

Determinants of health (e.g., race, sex, income, caregivers’ educational attainment) can advantageously or disadvantageously influence a child’s positioning within society as well as their developmental trajectories [4,26]. Marginalized children are less likely to acquire the care they need in healthcare settings that are discriminatory [27]. Female children are more likely to ascertain certain developmental milestones on average than male children, and vice versa [28]; female children are also more at risk of experiencing gender-based violence and inequities within educational and employment settings [29]. Additionally, other factors, such as cultural expectations, can influence how children interact with others in their early years and engage in different environments [30,31]. For example, cultures encouraging artistic expression may place significant emphasis on linguistic development, and those upholding gender stereotypes may observe differences in developmental domains due to gender expectations [30,32]. Tools that measure child development without evidence of cross-cultural applicability may produce erroneous developmental scores, referred to as cultural bias [30], and while tools are often adapted to overcome language barriers and aid in cultural applicability, adaptations may affect the reliability and validity of these measures [30]. Therefore, translated tools must also be completed in such a way that the questions continue to measure the intended outcome, undergoing their own psychometric analysis to ensure they are not only valid and reliable but also sensitive to cultural differences [33].

### 1.4. Assessment versus Screening Tools

It is important to note that tools can differ in their overall purpose. Screening tools (e.g., the Ages and Stages Questionnaire (ASQ)) may provide healthcare professionals with a preliminary understanding of whether children may be at risk of a certain developmental delay [8,34]. These tools often do not require any specialized training to be employed. They provide insight on whether a more comprehensive assessment is necessary but are not diagnostic [8,34]. On the other hand, assessment tools (e.g., Bayley Scales of Infant and Toddler Development (BSID)) provide more detailed understandings of developmental outcomes, allowing for diagnoses and subsequent treatment recommendations [8]. They often require specialized training prior to use and more time to complete, but they can provide a diagnosis that enables subsequent healthcare support [8]. Understanding the differences between the two is important when considering their use in clinical and research settings, as the former may not truly capture the development of a child and lead to incorrect diagnoses if used alone, while the application of the latter can be time-consuming and resource-intensive if unnecessary.

### 1.5. Purpose of the Study

Other researchers have completed reviews on existing screening and assessment tools for child development [35,36,37]; however, they do not provide a description of their use in different contexts (i.e., genetic, cultural, and perinatal factors) while also considering accessibility (e.g., cost). Because tools’ applicability in measuring child development can be impacted by cultural variability [38], genetic and inherited conditions [39], and other clinical/health groups [40], this comprehensive realist review aims to (1) provide a pragmatic compendium of literature evaluating the accessibility (i.e., cost, administration time, and training requirements), validity, and reliability of multidomain child development screening and assessment tools and (2) highlight the literature discussing their use in different cultural and clinical groups (applicability). Findings from this review can guide healthcare professionals in choosing the optimal tool when screening or assessing children’s development.

## 2. Methods

### 2.1. Review Methodology

This study employed an adaptation of pragmatic realist review methods to identify multidomain developmental child tools and to distinguish how, how well (e.g., psychometrics), for whom, and when these tools are used [41,42]. Searches were initiated in May 2020 and updated from December 2022 to January 2023, reviewing a large body of literature. While realist review methods excel at producing explanations, only tentative recommendations may be drawn [41]. Therefore, to enable more concise recommendations, tools were evaluated based on pre-specified rating criteria developed by our team after an examination of existing literature.

### 2.2. Search Strategy and Inclusion Criteria

To be included, tools were (1) described as multiple developmental domains (more than two to increase applicability), (2) still in use, and (3) designed for young children (newborns to five-year-olds). We first searched concepts including “early childhood development” AND “language”, “cognitive”, “mental”, “motor”, “communication”, AND “screening tool” or “psychometrics” on APA PsycInfo to identify child development tools, retrieving 496 evidence sources. Backward and forward searches helped identify other developmental tools. We then searched APA PsycInfo, MEDLINE, CINAHL, and ERIC with the names of identified tools, resulting in 4110 evidence sources. Backward and forward searches also supported the saturation of data collection. After this, we searched through gray literature via Google to collect other pertinent information about tools (e.g., costs and time to administer). We included evidence sources in English that provided general information about the included tools, examined their validity and reliability, or described their use in different contexts. We provide definitions of the subtypes of validity and reliability considered in this review (Table 2).

### 2.3. Data Collection and Analysis

All studies captured by the search were entered into individualized Excel spreadsheets designed for each tool. Three individuals (SK, JK, EP) independently participated in screening, first reviewing the titles and abstracts for eligibility. Studies that included validity and reliability analyses and described contextual applications were highlighted for each tool, and the data were then extracted onto the same spreadsheet. Two authors (SK, JK) completed data extraction and saturation with high agreement based on a preliminary test of 50 articles (December 2022 to April 2023). We excluded some studies and test manuals due to an inability to locate or access them despite our best efforts (an appropriate approach within realist review methods; [41]).

Tools were divided and compared in terms of their use in screening or assessment. Saturation was achieved when identifying up to three papers (excluding the test manual) with evidence of high validity or reliability for each respective type, with the most recent papers used first. We reviewed the cost, time, training, applicability, validity, and reliability of the included tools, initially rating them by their validity, reliability, and contextual application and then by their cost, time, and training; all the criteria led to an overall rating. However, validity, reliability, and contextual application largely contributed to the overall rating given the need for psychometric evidence to ensure accurate screening and assessment before considering accessibility. We developed rating ranges (i.e., low, moderate, and high) for costs, time to administer, and training to create a relatively fair distribution of the tools while also ensuring that ranges were sensibly based on the type of tool being examined (screening versus assessment).

For validity and reliability, a low rating indicated no evidence sources supporting high reliability and/or validity for each respective type beyond the test manual, a moderate rating indicated that there were two evidence sources supporting high reliability and/or validity for each respective type (along with the test manual, if available), and a high rating indicated that there was evidence from more than two publications (excluding the test manual) supporting high reliability and/or validity for each respective type. With a slightly more liberal approach compared to other literature, we included any reliability or validity estimates with correlation coefficients above 0.40 (moderate to high correlation; [46]) and estimates using other coefficients (e.g., Cronbach’s alpha, sensitivity, and specificity) above 0.70 (moderate to high estimates; [47,48]); we considered any values below these coefficients as a lack of evidence for validity or reliability and thus excluded these studies. Due to substantial similarity in versions of the same tool, we reported on tool validity and reliability based on more recent versions first. We also discussed the use of tools in different cultural and clinical groups in terms of available sources, but more detailed information can be found in Table S1 of Supplementary Materials.

## 3. Results

This review captured 41 tools that assessed multiple developmental domains. Of those, eight were removed due to being discontinued or no longer in use, including the ABILITIES Index [49], Cognitive Ability Scale [50], Developing Skills Checklist [51], Developmental Activities Screening Inventory [52], Developmental Observational Checklist System [53], Diagnostic Inventory for Screening Children [54], Gesell Developmental Schedules [55], and Miller Assessment for Preschoolers [56]. Therefore, we included 33 tools (Table 3). Despite using systematic methods in this realist review, applying important search concepts, and conducting backward and forward approaches in searches, we are aware and acknowledge that the review did not capture all existing screening and assessment tools for young children; nevertheless, we collected an impressively large body of literature on 33 young child development tools. Information including the domains assessed, age group, training and cost, administration, and use in different cultural and health groups for the included tools is provided (see Supplementary Materials, Table S1). Validity and reliability results are also provided (see Supplementary Materials, Table S2).

### 3.1. Comparing Screening Tools

#### 3.1.1. Comparing the Validity, Reliability, and Contextual Application of Screening Tools

For each respective form of reliability and validity, many screening tools were rated “low” (Table 4), implying that no evidence sources of validity or reliability were available. However, the ASQ consistently rated highly across all three forms of reliability and validity, providing strong psychometric evidence for the developmental screening of young children. Except for inter-rater reliability, the DIAL also rated highly on all other psychometric forms, reflecting its potential as another valid and modestly reliable screening tool. The DDST rated highly on all forms of reliability, suggesting that it is a reliable screening tool but less valid due to a lack of high evidence for structural, discriminant, or discriminative and predictive validity. All three tools also demonstrated use in a variety of clinical groups. The ASQ had studies completed among children with epilepsy, complex congenital heart disease, low birthweight, early gestational age, and cancer, whereas the DDST was used among children with sickle cell anemia and the DIAL among children with autism spectrum disorder. Other tools with lower validity and reliability also demonstrated evidence for use among highly specific clinical groups (e.g., the DAYC in conjunction with magnetic resonance detects cerebral palsy). Of the three tools, the ASQ and DDST demonstrated compelling evidence for use across several different cultures, whereas the DIAL lacked evidence for use in cultural groups outside the United States. All three tools (alongside other screening tools) demonstrated limited evidence for use in Central and South America, Africa, and Australia. While other screening tools showed use across different clinical and cultural groups, a lack of affordability, validity, and reliability limited further discussion of their use in healthcare settings.

#### 3.1.2. Screening Tool Cost, Time, and Training

Costs, administration times, and training required for screening tools are compared in Table 4. Screening tool costs ranged from free (R-PDQ) or a few cents (PEDS) to USD 877 (DIAL, although it can be used to screen multiple children at once). The time to administer screening tools ranged from less than five minutes (NDDS, PEDS, and PEDS DM) to one hour (DDST). Most screening tools required little to no training to administer and were usable among young children (i.e., ASQ, ADST, BRIGANCE, CDI, DAYC, DP, NDDS, PEDS, PEDS DM, and R-PDQ), indicating increased accessibility and applicability. The NDDS, PEDS, and PEDS DM rated highly on all three criteria, suggesting that they are affordable, quick to administer, and require little to no training.

#### 3.1.3. Combining the Results

When considering only cost, time to administer, and training, the NDDS, PEDS, and PEDS DM consistently rated highest; however, they achieved moderate to low scores across forms of reliability and validity. Conversely, because the ASQ rated consistently moderate on the former criteria and consistently high across the latter, it was rated as the most favorable screening tool based on this review. However, for use specifically in the United States and in larger child centers (e.g., classrooms), the DIAL was rated as a favorable tool because it can be used to screen several children at once, thus negating its higher cost. For organizations or centers prioritizing cheaper, quicker, and easily trainable screening tools over validity and reliability, the PEDS and PEDS DM were rated highly, with many translated versions.

### 3.2. Comparing Assessment Tools

#### 3.2.1. Comparing the Validity, Reliability, and Contextual Application of Assessment Tools

Compared to validity measurements, many assessment tools lacked high reliability (Table 5). However, the BSID consistently rated highly across all three forms of reliability and validity, providing strong psychometric evidence for assessing young children’s development. Except for structural, discriminant, or discriminative validity, the BDI rated highly on all psychometric forms, reflecting its potential use as another reliable and modestly valid assessment tool. The MSEL appeared to have moderate reliability to high validity, suggesting its close follow-up as an assessment tool in terms of validity and reliability. The use of all three tools in various clinical groups increased their applicability. BSID studies examined its use among children with congenital heart disease, early gestational age, infants exposed to HIV, and neurodiverse children, whereas the BDI and MSEL were more applicable for children with neurodiverse diagnoses and developmental delays. Some assessment tools demonstrated use among other clinical groups (e.g., CAT/CLAMS differentiates between global developmental delay and language problems). Of the three tools, the BSID demonstrated compelling evidence for use across numerous cultures, followed by the MSEL. NEPSY had many studies on validity and reliability; however, these studies included populations of children above five years of age. Central and South America, Africa, and Australia had limited studies on tool use.

#### 3.2.2. Assessment Tool Cost, Time, and Training

Costs, administration times, and training required for assessment tools are compared in Table 5. Assessment tool costs ranged from USD 70 (COR) to 1999 (GMDS). Administration times ranged from approximately 15 min (MSEL) to 2–3 months (COR). All assessment tools require trained professionals to administer them (e.g., clinicians and teachers); however, some only require an undergraduate degree/diploma (e.g., AEPS), whereas others require a post-graduate degree (e.g., BDI). Of all the assessment tools, only the CAT/CLAMS rated highly across all criteria; however, the GDO-R and the VABS also rated highly on cost and administration (information regarding training for these tools was unlocatable).

#### 3.2.3. Combining the Results

When considering only cost, time to administer, and training, the CAT/CLAMS, GDO-R, and VABS rated highest; however, these tools rated inconsistently across reliability and validity forms. While the BSID rated highly on the latter, low ratings on the former suggest a lack of accessibility for users. On the other hand, the MSEL rated relatively well across validity and reliability values, was used in some different cultural and clinical groups, and rated better than the BSID in terms of administration time, suggesting similar potential use. The BDI also showed high psychometric evidence (except moderate in structural, discriminant, or discriminative validity) and use in various clinical and cultural groups, and although rated similarly in administration time and training as the BSID and MSEL, it was less than half the cost of either tool. Therefore, based on this review, the BDI appears to be the most favorable assessment tool. However, for use specifically in the United States in Head Start programs, the COR may be a more suitable tool given evidence for its use in such programs. For organizations or centers prioritizing cheaper, quicker, and easily trainable assessment tools over validity and reliability, the CAT/CLAMS, GDO-R, and VABS are the most suitable tools.

## 4. Discussion

This adapted realist review aimed to provide a compendium of validity and reliability data on various multidomain developmental screening and assessment tools for young children, along with contextual information on their accessibility and applicability across different groups (i.e., cultural and clinical groups). Almost all tools had at least one study demonstrating one type of validity and reliability. Screening tools that cost the least and were the fastest to administer were the NDDS, PEDS, and PEDS DM, whereas the DIAL cost the most and the DDST required the most time to administer. Training requirements varied between screening tools, providing individuals in clinical, community, and research settings with options that fit their qualifications, resources, and employment settings. However, the DAYC, DDST, DIAL, DP, ESI-R, ESP, IDI, ITC, R-PDQ, and SDT had specific training and educational requirements, requiring more resources to be attained prior to conducting screening evaluations. The ASQ and DDST (along with BINS) demonstrated compelling evidence for use in different countries, and the ASQ, DDST, and DIAL were used in several clinical groups. The ASQ was identified as the most favorable screening tool; however, the DIAL may be preferred when screening larger groups of children in the United States. However, we do acknowledge that other tools may be preferred in different contexts (e.g., TLMQ in Iceland).

The CAT/CLAMS, GDO-R, and VABS were consistently the most affordable, least time-consuming, and least training-intensive of the assessment tools; however, these tools scored lower on validity and reliability, and only the VABS showed some cultural and clinical diversity group application. The BSID was the only assessment tool that scored highly across all forms of validity. However, the BDI scored high on all forms except for structural, discriminant, or discriminative validity, was applicable across diverse cultural and clinical groups, and was more accessible in terms of cost (34.6% of the cost of the BSID), suggesting the BDI as the most generally effective assessment tool. However, the COR may be preferred in the United States in Head Start programs due to evidence for its use in such settings, reflecting how other tools may be more effective in different contexts.

### 4.1. Validity across Screening and Assessment Tools

In general, screening tools lacked high validity, whereas assessment tools rated relatively highly across all examined forms. Only the ASQ and DIAL screening tools and the BSID assessment tool rated highly across all three types of validity; however, the BDI also rated highly except when scoring moderate on structural, discriminant, and/or discriminative validity. These findings suggest that these tools have been the most studied relative to the performance of other tools, in addition to the examination of their internal constructs.

Evidence of structural, discriminative, and discriminant validity can provide assurance to professionals (e.g., in healthcare or early childhood development settings) that the developmental tools they are using accurately screen/assess the intended outcomes or constructs [45]; however, based on this review, only 3/16 screening tools (i.e., ASQ, DIAL, ITC) had high structural, discriminant, and/or discriminative validity. Lack of such validity reveals an important gap in research and clinical knowledge, which threatens the accuracy of screening children [57]. Tools lacking this form of validity may not measure the intended construct, leading to false positives (or negatives) and erroneous referrals, preventing accurate diagnosis and treatment [57]. Most assessment tools (i.e., AEPS, BSID, COR, MSEL, NEPSY, VABS; 6/11), however, had high validity of this type, suggesting that if children are correctly screened for the developmental outcome of interest, several assessment tools exist with the ability to measure the outcome of interest and to compare clinically similar (or different) child groups. However, assessments require accurate screening; therefore, it is recommended that researchers further examine the structural, discriminant, and/or discriminative validity of screening tools to optimize the overall developmental evaluation process of children.

Concurrent and/or convergent validity correlatively examines outcomes with another tool (the gold standard in the case of concurrent validity; [57]). Most screening tools fell within the moderate to low category (16/22); therefore, more research is needed on their capacity to screen for children’s development relative to a more studied tool. When considering the implementation of child screening and assessments in different regions, evidence of concurrent and convergent validity of cheaper and less timely tools may provide an opportunity to overcome health disparities in impoverished areas [58], exemplifying the importance of such validity. Almost all assessment tools (8/11) captured in this review had high concurrent and/or convergent validity, potentially providing healthcare professionals with more options when assessing certain developmental outcomes (e.g., using the BDI instead of the BSID in Colombia or among children with neurodiverse diagnoses, saving USD 779 per child).

Predictive validity is particularly important in child development tools. Identification of plausible developmental milestones that should be attained given a child’s health and age allows healthcare professionals to track how a child might progress developmentally and may allow for a more integrative approach to developmental screening and assessment [59]. Only 4/22 screening (i.e., ASQ, BINS, DIAL, PEDS) and 4/11 assessment (i.e., BDI, BSID, CAT/CLAMS, GMDS) tools demonstrated high predictive validity, suggesting profound implications for the ability of healthcare professionals to predict children’s development over time. Because screening tools are cheaper, less timely, and less arduous to complete, predictive validity is particularly important in their application, as it can permit practitioners and clinicians to initially predict how children are developing and identify any underlying conditions sooner to prevent health sequalae from occurring [60]. This implies not only positive implications for public healthcare (e.g., lower healthcare costs and fewer resources toward assessments) but also for children and families, revealing the significant benefit of a primary preventive approach to child health.

### 4.2. Reliability across Screening and Assessment Tools

In general, both screening and assessment tools lacked high reliability. Only the ASQ and DDST screening tools and the BSID and BDI assessment tools rated highly across all three types of reliability forms, suggesting that these tools have well-developed internal constructs in addition to their reproducibility between two individuals (inter-rater reliability) or by the same person over time (intra-rater reliability).

To ensure that all the items of a child developmental screening tool are assessing a specific outcome (i.e., a developmental domain), studies on internal consistency reliability are required [57]. This is exemplified through assessments of adult mental health, where issues have arisen in differentiating between anxiety and depression items [61]. Only 4/22 screening (i.e., ASQ, DDST, DIAL, ITC) and 3/11 assessment (i.e., BDI, BSID, VABS) tools in this review had high internal consistency reliability; therefore, the items on tools rated moderate to low may not be reliably inferred as measuring the outcome of interest in its entirety [57]. Additional evaluations of internal consistency reliability are needed for tools classified as low to moderate.

Intra-rater (i.e., test–retest) and inter-rater reliability measurements provide information on the reproducibility of screening and assessment by the same individual or between individuals, respectively [57]. If a tool is difficult to administer (e.g., requires significant training), the accuracy of developmental assessments may differ between administrators, which in turn reduces the assessment’s accuracy [62]. Only 2/22 screening (i.e., ASQ, DDST) and 4/11 assessment tools (i.e., AEPS, BDI, BSID, MSEL) rated highly on inter-rater reliability in this review; the limited evidence for similar use between different individuals suggests the potential for differential classification as a function of the user [57]. It is also important that a professional can apply the same tool over time with similar results. Similarly, only 3/22 screening (i.e., ASQ, DDST, DIAL) and 2/11 assessment (i.e., BDI, BSID) tools rated highly on intra-rater reliability, which implies that other tools may not be understood or applied correctly when evaluating a child, leading to erroneous diagnoses. The busy environments and lack of resources in healthcare centers may explain the limited evidence of these two types of reliability [63]; however, their significance in ensuring appropriate child development evaluation cannot be overlooked and should be further investigated.

### 4.3. Contextualization across Different Groups

Most multidomain child development tools, whether screening or assessment, were utilized in North America, Europe, and Asia, requiring more evaluations in Central and South America, Africa, and Australia. Given this gap in use and evaluation, it is not surprising that some screening and assessment tools were specifically developed and tested for certain continents, such as the ADST in Australia, which reflects a solution (if psychometrically researched) to the lack of tools for certain cultural contexts. Culturally sensitive tools provide a means to overcome differences in cultural expectations, values, and languages, preventing cultural bias from affecting outcomes [64]. The ASQ and DDST were used as screening tools across many different cultural groups, which implies more culturally sensitive approaches to child development screening and subsequent referral for follow-up. The BSID and the DDST (although the DDST was not evaluated in Australia) had studies performed in at least one country on every continent, suggesting preliminary evidence for their use globally in assessing child development. Few tools have translated versions available in more than two languages, which eliminates their application within any other language groups. For tools that had translated versions, few had psychometric analysis to support their use among the specific languages they were translated to, potentially overlooking cultural differences, altering the meaning and descriptions of items deriving from the original instrumentation, and triggering feelings of stigma, blame, or shame among families through misinterpretations of translated questions and items [33,38]. For example, the PEDS had over 60 translations available; however, no psychometric evidence was available for a large majority of the translated versions. Children being screened or assessed with a tool unsuitable for their culture or language may result in an inaccurate representation of their developmental trajectories [64]. The development of revised versions for different cultures that are psychometrically assessed may allow healthcare professionals to overcome such challenges and ensure all children are receiving proper screening and assessments within professional settings despite cultural differences.

Children born preterm or at lower birthweights, along with those experiencing adverse antenatal conditions, are at risk of worsened developmental outcomes that may persist throughout their lifetime [20,40]. Similarly, children with certain genetic disorders or health conditions are likely to experience delays in achieving developmental milestones [65]. Some screening tools, such as the ADST and CDR, had no studies completed among these different contexts based on those captured by this review, suggesting that they are not applicable for screening or assessing children presenting with disabilities or health conditions. Conversely, the ASQ and DDST were used in several different clinical groups, suggesting sensitivity to the diverse developmental milestones of children with different disabilities. Many assessment tools were used among highly specific clinical child groups, such as the BSID for infants exposed to HIV, the CAT/CLAMS for differentiating between global development delay and language problems, the DDST for children with sickle cell anemia, the DAYC-2 to identify cerebral palsy (in conjunction with magnetic resonance), and the NEPSY among children with Asperger’s disorder. Nevertheless, it is imperative that more studies be conducted on specific developmental disabilities to provide healthcare professionals with diverse options (e.g., cost). Further, more studies are required to examine how different cultural and clinical contexts may intersect and affect developmental trajectories concomitantly.

### 4.4. Future Research

Intra-rater and inter-rater reliability studies comprise an area in which future researchers could focus in order to ensure reproducibility between the same individual or between two different individuals, respectively. Most of the studies provided evidence for tool use in North America, Europe, and Asia, but more studies are needed on their performance in Africa, Australia, and Central and South America to provide professionals with more options and comfort in applying tools among different cultural groups. Though some tools may be designed specifically for certain countries (e.g., TLMQ in Iceland), these tools require much more psychometric analysis to ensure that they are measuring the intended developmental domain. Not all tools will be designed for every purpose or group; however, ensuring that several valid and reliable tools exist that consider the diversity of children (i.e., culture and health) can encourage more accessibility, equity, and optimal developmental screening and assessment by healthcare professionals to improve lifelong outcomes in children. In relation to this, researchers should focus on collecting available evidence and conducting assessments of measurement invariance (which ensures that a construct has the same meaning across different sociodemographic groups [66]) and item response theory (which helps to identify interindividual variation and reduce scales to shorter versions when possible [67]) to ensure tools are adequate across different identity groups and to limit response burden.

### 4.5. Strengths and Limitations

Although the search was comprehensive, the test manuals for certain tools were unidentifiable or inaccessible, and it is possible that some tools had validity and reliability data not found in the search. However, this may not necessarily be a limitation of the study itself but rather a reflection of the difficulty in accessing important data and information that researchers need to adequately conduct child development research. Though realist review methods allow for saturation to be achieved and permit exploration to identify what tools are best and for whom, under various circumstances (e.g., culturally diverse populations; [41]), employing adapted realist review methods may hinder the possibility of reproducibility. We did nevertheless attempt to be as clear as possible in our methods to allow for transparency and for other researchers to understand how this study was completed. Included studies were also not critically appraised as this was beyond the scope of this paper, but this review provides researchers, healthcare professionals, and policymakers with a compendium of data on each tool, facilitating access to validity, reliability, and contextualized analyses and the identification of the most appropriate tool for a healthcare professional under a certain circumstance. Further, a compelling body of literature was reviewed, providing important information and data on over 30 screening and assessment tools for young children’s development.

## 5. Conclusions

This adapted realist review developed a compendium of multidomain developmental screening and assessment tools for young children to describe their validity, reliability, accessibility, and applicability in various contexts. Almost all tools had studies on at least one form of validity and reliability. Often, researchers cite evidence for reliability or validity from other studies, which may not be fully reflective of the tool’s psychometric capacity in their study population. The ASQ appeared to be the most valid, reliable, and contextually applicable screening tool, with moderate to high ratings across affordability and accessibility; however, evidence from this review may favor the use of the DIAL in larger child groups (e.g., preschool daycares) in the United States. On the other hand, findings from this review highlight the use of the BDI as an assessment tool, as it performed almost as well as the BSID at 36% of the cost. However, some tools may complement each other in certain contexts; more specifically, when considering specific clinical groups, one could consider using the ASQ or the BDI with other tools that have psychometric evidence among specific clinical groups. For example, the ASQ could be used alongside the DAYC when screening development among children with cerebral palsy, while the BDI could be used alongside the GMDS when assessing development among children with Down syndrome. In terms of culture, some tools do demonstrate potential for unique use in specific regions (e.g., ADST in Australia), warranting further consideration. It is imperative to examine the intersections between the determinants of health, genetics, and the caregiving environment in the context of multidomain developmental screening and assessment so that healthcare professionals are properly equipped to evaluate young children’s development and optimize child and family health.

## Figures and Tables

**Table 1:** Definitions of commonly cited developmental domains [6].

Developmental Domain	Definition
Multidomain tools	Refers to three or more developmental domains.
Physical	Refers to children’s physical health and development (e.g., growth, muscle movement and coordination, nutrition, and physiological functioning). Muscle movement and coordination are often subdivided into two categories: (1) gross motor (those pertaining to larger muscle movements) and (2) fine motor (those pertaining to smaller, more refined muscle movements).
Cognitive	Refers to the cognitive level and functioning of the child, including but not limited to executive functioning, impulse control, reasoning, learning competencies, and problem solving. The ability to adapt and self-regulate also falls under this developmental domain; however, it may be considered a fifth developmental domain.
Social–emotional	Refers to the ability of children to regulate their emotions, engage in social environments, build/maintain relationships, and have good overall social and emotional health. Behavioral concerns often manifest when there are underlying social–emotional health issues (e.g., mental health).
Language	Refers to the ability to communicate, recognize and differentiate sounds, and have literacy skills.

**Table 2:** Definitions of different validities and reliabilities considered in this review.

Psychometric Term	Definition
Structural, discriminant, and discriminative validity	Subtypes of construct validity reflecting the ability of a tool to measure what it is actually designed to measure. Structural validity ensures that a tool is measuring its hypothesized construct. Discriminant validity measures whether constructs that are supposed to be unrelated are in fact unrelated. Discriminative validity measures whether a tool can differentiate between groups that are expected to differ on the construct of interest [43].
Concurrent and convergent validity	Though convergent validity is a subtype of construct validity and concurrent validity is a subtype of criterion validity, both provide a measure reflecting a tool’s performance relative to another tool designed to measure the same concept. Concurrent validity is assessed with a criterion (the gold standard), and convergent validity is utilized in the absence of a gold standard [43]. While conducting this review, consensus appeared to be lacking on what tools comprise the gold standard for evaluating child development. Therefore, we grouped these categories together.
Predictive validity	A subtype of criterion validity reflecting a tool’s performance relative to a criterion’s (the gold standard) performance on an outcome that is in the future [43].
Internal consistency reliability	A measure describing how consistently the items on a tool measure a variable or behavior of interest within a short time period, reflecting a ratio of variance of true versus observed scores [44].
Intra-rater reliability	A measure considering the scores retrieved from a measure completed by the same individual at two different time points [45].
Inter-rater reliability	A measure comparing the scores retrieved from a measure by two different individuals, examining their agreement [45].

**Table 3:** Included tools (n = 33) and their abbreviations.

Tool Name	Abbreviation	Tool Name	Abbreviation
**Screening Tools (n = 22)**		**Screening Tools Continued (n = 22)**	
Ages and Stages Questionnaire	ASQ	Parents’ Evaluation of Developmental Status Developmental Milestones	PEDS DM
Australian Developmental Screening Test	ADST	Preschool Developmental Assessment Scale	PDAS
Bayley Infant Neurodevelopmental Screener	BINS	Revised Denver Prescreening Developmental Questionnaire	R-PDQ/PDQ-II
BRIGANCE Screens III	BRIGANCE	Shoklo Developmental Test	SDT
Child Development Inventory	CDI	Toddler Language and Motor Questionnaire	TLMQ
Child Development Review	CDR	**Assessment Tools (n = 11)**	
Copenhagen Infant Mental Health Screening	CIMHS	Assessment, Evaluation, and Programming System for Infants and Children	AEPS
Developmental Assessment of Young Children	DAYC	Battelle Developmental Inventory	BDI
Denver Developmental Screening Test	DDST	Bayley Scales of Infant and Toddler Development	BSID
Developmental Indicators for the Assessment of Learning	DIAL	Capute Scales (Cognitive Adaptive Test/Clinical Linguistic and Auditory Milestone Scale)	CAT/CLAMS
Developmental Profile	DP	Child Observation Record (COR) Advantage	COR
Early Screening Inventory—Revised	ESI-R	Comprehensive Developmental Inventory for Infants and Toddler	CDIIT
Early Screening Profiles	ESP	Gesell Developmental Observation Revised	GDO-R
Infant Development Inventory	IDI	Griffiths Mental Development Scales	GMDS
Infant–Toddler Checklist	ITC	Mullen Scales of Early Learning	MSEL
Nipissing District Development Screen (Looksee Checklist)	NDDS	NEPSY: A Developmental Neuropsychological Assessment	NEPSY
Parents’ Evaluation of Developmental Status	PEDS	Vineland Adaptive Behavior Scales	VABS

**Table 4:** Screening tool (n = 22) comparison of cost, administration time, training, and psychometrics.

	Cost (Low = Costs Higher than USD 400, Moderate = Costs between USD 100 and 400, High = Costs Lower than USD 100)		Administration Time (Low = Time to Complete Is More than 30 min; Moderate = Time to Complete Is 10 to 30 min; High = Time to Complete Is Less than 10 min)		Training Required (Low = Professional with Formal Education and Training (e.g., Specific Degrees); Moderate = Some Training (e.g., Working with Children) and a Professional or Supervised Caregiver; High = Little to No Training to Administer)	
Low	ADST, BRIGANCE, DAYC, DDST, DIAL, DP, ESP		DDST, DIAL, DP, ESP		DAYC, DDST, DIAL, DP, ESI-R, ESP, SDT	
Moderate	ASQ, BINS, CDI, ESI-R, ITC		ASQ, ADST, BINS, BRIGANCE, CDI, CDR, DAYC, ESI-R, R-PDQ, SDT		ADST, IDI, ITC, R-PDQ	
High	CDR, IDI, NDDS, PEDS, PEDS DM, R-PDQ		ITC, NDDS, PEDS, PEDS DM		ASQ, BINS, BRIGANCE, CDI, CDR, CIMHS, NDDS, PEDS, PEDS DM, TLMQ	
	**Internal Consistency Reliability**	**Intra-Rater Reliability**	**Inter-Rater Reliability**	**Structural and Discriminant/Discriminative Validity**	**Concurrent/Convergent Validity**	**Predictive Validity**
Low	ADST, BRIGANCE, CDR, CIMHS, DP, ESI-R, ESP, IDI, NDDS, PEDS, PDAS, R-PDQ, SDT, TLMQ	ADST, BRIGANCE, CDI, CDR, CIMHS, DP, ESI-R, IDI, NDDS, PDAS, R-PDQ, SDT, TLMQ	ADST, BINS, BRIGANCE, CDI, CDR, CIMHS, DAYC, DP, ESI-R, ESP, IDI, NDDS, PEDS DM, PDAS, SDT, TLMQ	ADST, BRIGANCE, CDR, CIMHS, DDST, DP, ESI-R, ESP, IDI, NDDS, PEDS, PEDS DM, PDAS, R-PDQ, SDT, TLMQ	CIMHS, DAYC, DP, ESI-R, IDI, NDDS, PDAS, SDT, TLMQ	ADST, BRIGANCE, CDI, CDR, CIMHS, DAYC, DP, ESI-R, ESP, IDI, NDDS, PEDS DM, PDAS, R-PDQ, SDT, TLMQ
Moderate	BINS, CDI, DAYC, PEDS DM	BINS, DAYC, ESP, ITC, PEDS, PEDS DM	DIAL, ITC, PEDS, R-PDQ	BINS, CDI, DAYC	ADST, BINS, BRIGANCE, CDR, ITC, PEDS, PEDS DM	DDST, ITC
High	ASQ, DDST, DIAL, ITC	ASQ, DDST, DIAL	ASQ, DDST	ASQ, DIAL, ITC	ASQ, CDI, DDST, DIAL, ESP, R-PDQ	ASQ, BINS, DIAL, PEDS

**Table 5:** Assessment tool (n = 11) comparison of cost, administration time, training, and psychometrics.

	Cost (Low = Costs Higher than USD 1000, Moderate = Costs between USD 400 and 1000, High = Costs Lower than USD 400)		Administration Time (Low = Time to Complete Is More than 60 min; Moderate = Time to Complete Is More than 45 to 60 min; High = Time to Complete Is around 45 min or Less)		Training Required (High = Requires Post-Graduate Degree and Specific Courses; Moderate = Requires an Undergraduate Degree and Specific Training; Low = Requires Either an Undergraduate Degree or Specific Training to Administer)	
Low	BSID, GMDS, MSEL, NEPSY		AEPS, BDI, BSID, COR, GMDS		BDI	
Moderate	AEPS, BDI		CDIIT, MSEL		COR	
High	CAT/CLAMS, COR, GDO-R, VABS		CAT/CLAMS, GDO-R, NEPSY, VABS		AEPS, BSID, CAT/CLAMS, MSEL	
	**Internal Consistency Reliability**	**Intra-Rater Reliability**	**Inter-Rater Reliability**	**Structural and Discriminant/Discriminative Validity**	**Concurrent/Convergent Validity**	**Predictive Validity**
Low	AEPS, CAT/CLAMS, CDIIT, GDO-R, GMDS, NEPSY	AEPS, CAT/CLAMS, COR, CDIIT, GDO-R, GMDS, NEPSY	CAT/CLAMS, COR, CDIIT, GDO-R, NEPSY, VABS	CAT/CLAMS, CDIIT, GDO-R, GMDS	CDIIT, GDO-R	AEPS, COR, CDIIT, GDO-R, VABS
Moderate	COR, MSEL	VABS, MSEL	GMDS	BDI	AEPS	NEPSY
High	BDI, BSID, VABS	BDI, BSID	AEPS, BDI, BSID, MSEL	AEPS, BSID, COR, MSEL, NEPSY, VABS	BDI, BSID, CAT/CLAMS, COR, GMDS, MSEL, NEPSY, VABS	BDI, BSID, CAT/CLAMS, GMDS, MSEL

## Data Availability

No new data were created or analyzed in this study. Data sharing is not applicable to this article.

## Supplementary Materials

The following supporting information can be downloaded at https://www.mdpi.com/article/10.3390/children11060745/s1, Table S1: Information about screening and assessment tools; Table S2: Reliability and validity of screening and assessment tools.
