# Supplementary material for: Validity, Reliability, Accessibility, and Applicability of Young Children’s Developmental Screening and Assessment Tools across Different Demographics: A Realist Review
Source: Children (Basel). 2024 Jun 18;11(6):745. doi: 10.3390/children11060745 (PMC11201752; doi:10.3390/children11060745)
Supplement: Supplementary file 1 [file children-11-00745-s001.zip › children-3056359-supplementary.pdf]

Table S1.

Information about screening and assessment tools

Tool Name	Domains	Age Group	Training and Cost	Administration	Cultural Diversity	Health Group Diversity
**Screening Tools (n=22)**						
Ages and Stages Questionnaires (ASQ)<br><br>Newest Edition: 3rd Edition<br><br>(Jane Squires et al., 2009)	Fine and gross motor, personal-social, communication, problem solving	1 to 66 months (prematurity, i.e., ≤ 37 weeks gestation) is adjusted for with children ≤ 2 years)	-Cost: $295 USD for starter kit (i.e., user guide and questionnaires that can be photocopied)<br>-Training: Appears to require minimal training; users read the user guide, and additional training is available online	-Quick completion: approximately 10-15 minutes for parents to complete and 2-3 minutes to score (scores range from 0 to 10)<br>-Parent reported questionnaire; parents attempt developmental skills with child	- Numerous cultural adaptations (e.g., Canada Mohawk First Nation, India, Korea) and language translations (e.g., Afrikaans, Mandarin; El-Behadli et al., 2015; Rousseau et al., 2021; Small et al., 2019)	Benefit demonstrated for diverse (e.g., children born premature and/or with low birthweight, children with epilepsy, complex congenital heart disease, cancer, children living in foster care) groups (Marks et al., 2019).
Australian Developmental Screening Test (ADST)<br><br>(Burdon, 1993)	Personal-social, language, cognition, fine and gross motor	Children birth to 5 years of age	-Cost: ~$480 USD (ADST Test Kit and forms need to be purchased)<br>-Training: No formal training required but designed for professionals who work with children (e.g., healthcare, educators)	-Quick completion requiring 15-20 minutes<br>-Play-based observations where a child is asked to perform a skill<br>-Screening provides a developmental age: delay of ≥ 4 months in one domain or ≥ 2 or more months in > 2 domains requires a formal developmental assessment (Morris et al., 2012; Zwi et al., 2016)	-Developed and standardized in Australia and also administered to refugees in Australia (Morris et al., 2012)	Limited publications found prevents understanding of group applicability.
Bayley Infant Neurodevelopmental Screener (BINS)<br><br>(Aylward, 1995)	Sensory development (e.g., auditory, visual), expressive (e.g., verbal), motor, cognitive processes	3 to 24 months	-Cost: $306 USD for entire kit<br>-Training: Appears to require limited training (e.g., read manual, familiarity with test); educational considerations can be found at www.pearsonacademy.com	-10-15 minutes to administer<br>-Administered by professional<br>-Test comprised of 11-13 items scored as optimal or non-optimal and child identified at low, moderate, or high risk for developmental delay	-A review on translation can be accessed (El-Behadli et al., 2015)<br>-Validated in Turkey (Soysal et al., 2014), South America (Guedes et al., 2011), and South Africa (Rodriguez et al., 2020)	Used to assess children at risk for developmental and neurodevelopmental delays. It can also be used to assess high-risk infants such as those admitted to intensive care units (Aylward, 1995).
BRIGANCE Screens III (BRIGANCE)<br><br>(Brigance & French, 2013)	Physical development (e.g., motor skills), language development, adaptive behavior (for infant and toddlers), academic skills,	0 months to end of first grade	-Cost: ~$529 USD<br>-Training: No qualifications required to administer (technical manual	-10-15 minutes to administer and score<br>-Scores compared to age-appropriate cut-offs	-Validity and reliability found in North America (Dempsey et al., 2016; Frisk et al., 2009) and translated to Spanish	Used for children who may have potential development delays or children who are gifted or academically talented

	cognitive development (for 2 years to first grade)		can also be accessed)		(El-Behadli et al., 2015)	(Brigance & French, 2013).
Child Development Inventory (CDI)<br><br>(Ireton, 1992)	Social, self-help, gross and fine motor, expressive language, language comprehension, letters, numbers, general development	1 to 6 years of age or for older children functioning at 1-to-6-year range	-Cost: $150 USD for CDI starter set (e.g., manual, booklets) (see https://childdevelopmentreview.com)<br>-Training: No formal training required	-15-30 minutes to administer, 10 minutes to score<br>-Parents answer yes/no questions. Scores compared to age norms. Scores < 30% or 25-30% below age line defined as "developmentally delayed" or "borderline development" (Ireton, 1992)	-Normed in the US with almost exclusively (95%) white sample (Ireton, 1992)<br>-Chinese and French version available (Duyme et al., 2011)	Validity provided for children deemed high risk (e.g., low birth weight; Doig et al., 1999; Montgomery et al., 1999) and those with autism spectrum disorder (Fulton & D'Entremont, 2013).
Child Development Review (CDR)<br><br>(Ireton, 1990)	Social, self-help, gross and fine motor skills, language	18 months to kindergarten	-Cost: $90 USD for questionnaire and manual<br>-Training: No qualifications to administer parental report<br>-Exemplar accessible at: https://childdevelopmentreview.com/healthcaretools/cdr-parent-questionnaire	-Up to 20 minutes to administer<br>-Parent completes 6 questions and 26 possible problems checklist<br>-Child development chart ~~of the child development review~~ is used by professional to classify child as typically developing, borderline, or delayed (Ireton, 1996)	-Validity in the US (Voigt et al., 2007)<br>-Translated into Spanish and Russian (El-Behadli et al., 2015)	Limited publications found prevents understanding of group applicability.
Copenhagen Infant Mental Health Screening (CIMHS)<br><br>(Ammitzbøll et al., 2016)	Eating, sleep, emotion/expression, curiosity & interest, attention, motor, social communication & interaction, language	8 to 10 months	-Cost: Unable to locate source for purchase<br>-Training: Appears to require minimal training, but administered by community health nurses	-Appears to be a quick administration but timing not found<br>-24 item questionnaire administered by nurse<br>-Parent report and nurses' observations	-Assessed in Denmark (Ammitzbøll et al., 2016)	Limited publications found prevents understanding of group applicability.
Developmental Assessment of Young Children (DAYC)<br><br>Newest Edition: 2nd Edition<br><br>(Voress et al., 2013)	Cognition, communication, social-emotional, physical development, adaptive behavior	Birth to 5 years	-Cost: $636 USD for kit<br>-Training: Relevant degree/knowledge (e.g., healthcare, psychologist) required<br>www.pearsonclinical.ca	-10-20 minutes per domain (approximately 1.5 hours for all domains)<br>-Assessment involves observations, caregiver interviews, and direct assessment<br>-Items scored as passed (1) or not passed (0)	-Validity in Jordan (Rawan M Abu Saleh & Jamil M Smadi, 2017) and the US (Swartzmiller, 2014)	In conjunction with magnetic resonance imaging, used to detect cerebral palsy (Novak et al., 2017).
Denver Developmental	Personal-social, fine and gross	1 month to 6 years	-Cost: $407 USD for kit, forms, and manual	-20-60 minutes to administer	-21 translated languages (El-Behadli et al., 2015) and used	Used widely among groups and those with low income or children

Screening Test (DDST)<br><br>(Frankenburg et al., 1992)	motor/adaptive, language		-Training: Training required by a professional with a background in related field (e.g., teacher, clinician)	-Professional observations but primary caregiver can report on child’s typical responses	worldwide; for example, validity in Africa and Asia (Mendonça et al., 2016) and Colombia (Rubio-Codina et al., 2016)	with sickle cell anemia (Fleming, 1981; Schatz et al., 2008).
Developmental Indicators for the Assessment of Learning (DIAL)<br><br>Newest Edition: 4th Edition<br><br>(Mardell & Goldenberg, 2011)	Motor, language, concepts, self-help, social development	2.5 years to 5 years 11 months	-Cost: $877 USD for complete print kit<br>-Training: Relevant degree/knowledge (e.g., healthcare, psychologist) for site coordinator (www.pearsonassessments.com)	-30-45 minutes to complete<br>-Can screen several children at once using stations (e.g., interact with toys)<br>-Items scored from 0-4<br>-Parent/teacher questionnaires for social and self-help	-Standardized and published in US (Coughlan, 2015; Katz, 2016)<br>-English and Spanish versions available.	Validity with diverse groups (e.g., autism, developmental delay; Coughlan, 2015).
Developmental Profile (DP)<br><br>Newest Edition: 4th Edition<br><br>(Alpern, 2020)	Physical, adaptive, social-emotional, cognitive, communication	Birth to 21 years, 11 months	-Cost: Complete print kit $492 USD<br>-Training: Bachelor’s degree (e.g., psychology) required and familiarity with testing	-20-40 minutes to complete<br>-Interdisciplinary approach to assessment, including: parent or caregiver interview, clinician rating, teaching checklist, and parent checklist (Grade 5 reading level)	-Standardized in US (Alpern, 2020)	DP-4 administered to children with and without clinical diagnoses (e.g., autism spectrum disorder, developmental delay; Alpern, 2020).
Early Screening Inventory – Revised (ESI-R)<br><br>(Meisels et al., 2008)	Sensory development and adaptive (e.g., visual motor), language, cognition, gross motor	ESI-Preschool: 3 to 4.5 years<br>ESI-Kindergarten): 4.5 to 6 years	Cost: $231 USD for full kit<br>Training: Education requirements (e.g., healthcare, psychology) (www.pearsonclinical.ca)	-15-20 minutes to complete<br>-Individually administered<br>-Examiner observations and parent questionnaire	-Tested in US (Meisels et al., 1993)<br>-Spanish version available	Limited publications found prevents understanding of group applicability.
Early Screening Profiles (ESP)<br><br>(Harrison et al., 1990)	Cognitive, language, motor, and self-help, social profiles	2 years to 6 years 11 months	-Cost: $537 USD for full kit<br>-Training: Used by professionals (e.g., education, medical). www.pearsonassessment.com	-15-40 minutes to complete<br>-Surveys completed by parents or teachers and direct testing	-Standardized in US (Snow, 1995) and validity assessed in Canada (Frisk et al., 2009)	Designed to identify children with developmental disabilities or those who may be gifted (Snow, 1995).
Infant Development Inventory (IDI)<br><br>(Ireton, 1994)	Social, self-help, gross and fine motor, language	Up to 18 months	-Cost: $55 USD for IDI<br>-Training: A professional completes the assessment using an interpretative schematic on the tool	-No time provided for length of assessment, however, can be shortened if professional reviews parental report prior to observation<br>-Caregiver provides information pertaining to child’s behaviour then	-Appears to be normed in a US sample (https://childdevelopmentreview.com/)	IDI used to identify children with developmental delay versus those without and used in a sample with low-birth-weight infants. (https://childdevelopmentreview.com/).

			(https://childdevelopmentreview.com/)	professional directly examines baby<br>-Social development is mainly provided by parent		
Infant-Toddler Checklist (ITC)<br><br>(Wetherby & Prizant, 2002)	Language (i.e., sounds, words), symbolic (i.e., understanding, object use), and social (i.e., emotion and eye gaze, communication, gestures) composites	6 to 24 months	-Cost: ITC and scoring CD-ROM = $100 USD<br>-Training: Used by clinicians and is a subtest of the Communication and Symbolic Behavior Scales Developmental Profile™ Child assessment www.brookespublishing.com	-5-10 minutes to complete<br>-Parent reported with 24 developmental questions (scored as not yet, sometimes, often)<br>www.brookespublishing.com	-Normed in US and tested in various countries such as Taiwan (Lin & Chiu, 2014), Sweden (Fäldt et al., 2021), and Australia (Eadie et al., 2010)	Initially developed from a group of high-risk infants in a perinatal follow-up clinic in the United States.
Nipissing District Development Screen (NDDS); Now Looksee Checklist<br><br>(Nipissing District Developmental Screen Intellectual Property Association, 2000)	Sensory (e.g., visual, hearing), communication, gross and fine motor, cognitive, social-emotional, and self help	1 to 72 months	-Cost: $25 USD for all checklists in a convenient pocketbook<br>-Training: No training required	-Administration time < 5 minutes<br>-Simple "yes" or "no" questionnaire<br>-Parent-reported 17 item survey<br>-Scored using flags (i.e., 1 flag means child does not meet 1 milestone)(Cairney et al., 2016)	-Validity in Canada (van den Heuvel et al., 2016)<br>-Available in more than 10 languages (www.lookseechecklist.com)	Used in Ontario, Canada for children with mild and severe developmental delays (Cairney et al., 2016).
Parents' Evaluation of Developmental Status (PEDS)<br><br>(Glascoe, 1998)	Global/cognitive, speech/expressive language, receptive language, behavior, social-emotional, school, self-help, fine motor, gross motor, and others such as medical or sensory concerns	Birth to 7 years, 11 months	-Cost: $0.39 USD per visit<br>-Training: Limited to no training required to complete (https://pedstest.com/AboutOurTools/LearnAboutPEDS/IntroductionToPEDS.html)	-Requires < 5 minutes (< 2 minute to complete and score if performed as an interview)<br>-Parents complete a 10-question survey that is written at a Grade 4-5 reading level<br>-The assessment categorizes children as low, moderate, or high risk for developmental problems	-Used in more than 100 countries and translated in more than 60 languages (www.pedstestonline.com)	Used in various contexts such as in clinics, during community-based assessments, and among children with autism spectrum disorder (Doove et al., 2019; Sheldrick et al., 2020; Wiggins et al., 2014).

Parents' Evaluation of Developmental Status Developmental Milestones (PEDS DM)<br><br>(Kyle B Brothers et al., 2008)	Fine & gross motor, expressive & receptive language, self-help, social-emotional skills (reading and math included for 4–8-year-old children)	Birth to 7 years, 11 months	-Cost: $0.30 cents USD per visit<br>-Training: Limited to no training required to complete (https://pedstest.com/AboutOurTools/LearnAboutPEDSDM/)	-Requires < 5 minutes to complete<br>-Parents answer 6-8 items: each item is related to a separate developmental domain<br>-Direct child administration can be performed<br>-Grade 2 readability	-Validity found in US (Kyle B Brothers et al., 2008) and Jordan (Mattar & Arouri, 2017)<br>-60 translations available (www.pedstest.com)	Other screeners (e.g., for autism spectrum disorder) in supplemental measures and assessment level version for neonatal intensive care and child find programs.
Preschool Developmental Assessment Scale (PDAS)<br><br>(Leung et al., 2011)	Cognitive, social, language, gross and fine motor, visual perception, literacy, numeracy	3 years 4 month to 6 years 3 months (based on a study) (Leung et al., 2011)	-Cost: Unable to locate cost<br>-Training: Unable to locate training information	- Length of time to complete assessment not provided<br>-Children assessed by PDAS trained professional (e.g., research assistant)	-Four districts in Hong Kong (Leung et al., 2011)	Limited publications found prevents understanding of group applicability.
Revised Denver Prescreening Developmental Questionnaire (R-PDQ); also called PDQ-II<br><br>(Frankenburg et al., 1987)	Personal-social, fine and gross motor, language	Birth to 6 years	-Cost: Free with manual and test forms online (see:<br>-Training: Requires training https://www.pediatric-kidmed.com/patient-forms)	-10-20 minutes for administration and scoring<br>-Parent reported questionnaire and scored by professionals<br>-If PDQ suggests ≥ 2 development delays, child requires further evaluation	-Validity in the US (Frankenburg et al., 1987) and Canada (Karam et al., 2015)	Limited publications found prevents understanding of group applicability.
Shoklo Developmental Test (SDT)<br><br>(Haataja et al., 2002)	Cognitive functions, motor milestones, social development, speech	3 to 12 months	-Cost: Unable to locate information on cost<br>-Training: Requires a trained person to perform (e.g., pediatric neurologist)	-Requires 25 minutes to administer and score by a trained person (Haataja et al., 2002)<br>-Caregiver provides some report on speech and social ability, in addition to trained examiners observations across all domains (Haataja et al., 2002)<br>-Scored on a pass or fail basis (Haataja et al., 2002)	-Infants regarded as typically developing from a Karen refugee camp (Shoklo or Maela) displaced from the Thai-Burmese border and from London (Haataja et al., 2002)	Limited publications found prevents understanding of group applicability; however, created for resource poor settings (Haataja et al., 2002).
Toddler Language and Motor Questionnaire (TLMQ)<br><br>(Gudmundsson, 2015)	Gross & fine motor, language expression, comprehension, personal-social, self help	15 to 38 months	-Cost: Unable to locate information on cost<br>-Training: Requires no formal training to complete (e.g., completed by mother; Gudmundsson, 2015)	-Length of time to complete assessment not provided<br>-Reported by primary caregiver which is labelled as mother in the initial publication (Gudmundsson, 2015)<br>-Includes 144 items across the five subtests	-Standardized in Iceland (Gudmundsson, 2015)	Limited publications found prevents understanding of group applicability.
**Assessment Tools (n=11)**						

Assessment, Evaluation, and Programming System for Infants and Children (AEPS)<br><br>Newest Edition: 3rd Edition<br><br>(Johnson & Macy, 2019)	Math, literacy, adaptive, cognitive, fine motor, gross motor, social-emotional, social-communication	Birth to 6 years of age	-Cost: $499 USD for Complete Kit 3rd Edition<br>-Training: Requires training and administration by health professionals (e.g., early childhood educations, psychologists); curriculum-based criterion assessment	-Requires 30-120 minutes<br>-AEPS trained professionals (e.g., teachers, therapists) with child development knowledge administer test (e.g., naturalistic observations, direct testing, family report), utilizing a 3-point scale (2 = mastery, 1 = emerging skill, 0 = not yet)	-Has been published in English, Spanish, Canadian French, Finnish, Korean, and Traditional Chinese (Johnson & Macy, 2019)	Initially developed for children with disabilities but also utilized for typically developing children.
Battelle Developmental Inventory (BDI)<br><br>Newest Edition: 3rd<br><br>(Newborg, 2005)	Social-emotional, adaptive, motor, cognitive, and communication, math, literacy	Birth to 7 years, 11 months (3 years 6 months to 7 years 11 months for the literacy and mathematics domains)	-Cost: $413 for full developmental screening kit (www.riversideinsights.com)<br>-Training: Must be registered psychologists with post-graduate training in psychology (or those with suitable training) to purchase	-60-90 minutes for entire test, 5-10 minutes per subtest (there is also a BDI screener which requires 10-30 minutes)<br>-Assessments occur with structured methods, observations, and/or caregiver/teacher interview<br>-Scoring (range from 0 = not yet emerged skill to 2 = fully emerged) occurs by hand or via data manager<br>-See test review for more information (Alfonso et al., 2010)	-Standardized BDI written in English and translated to Spanish<br>-Utilized in the United States (US), Spain, Mexico, Russia, Israel, Pakistan, Chile, Colombia, Brazil, and Canada (Cunha et al., 2018)<br>-Validation also published in Colombia (Rubio-Codina et al., 2016), Taiwan with Mandarin version of BDI-2 (Ma, 2012), and Canada with BDI Screening Inventory (Frisk et al., 2009)	Studies on children with neurodiverse diagnoses (e.g., autism) and developmental delays found high specificity in identifying speech language delays (.75), motor delays (.80), developmental delays (.83) cognitive delays (.86), and autism (.91) (Hilton-Mounger, 2011).
Bayley Scales of Infant and Toddler Development (BSID)<br><br>Newest Edition: 4th<br><br>(Bayley & Aylward, 2019)	Cognitive, language, motor, social-emotional, adaptive	4th Edition: 16 days to 42 months	-Cost: Complete digital kit = $1192 and complete print kit = $1325<br>-Training: Training required via courses and a training consultant	-30-90 minutes for full assessment (dependent on child age)<br>-Children assessed by a trained professional with 3 scales (cognitive, language, motor) comprised of interactions and activities<br>-Social-emotional and adaptive behavioral reported by the primary caregiver.<br>See: www.pearsonacademy.com	-BSID standardized in the US (Albers & Grieve, 2007) and validity assessed worldwide: e.g., Bangledesh, Brazil, India, Nepal, Pakistan, Peru, and South Africa (Pendergast et al., 2018), Sweden (Månsson et al., 2019), Denmark (Krogh & Væver, 2019), Taiwan (Lin et al., 2020), Singapore (Goh et al., 2017), Vietnam (Nguyen, 2017), and	Extensively used with diverse groups, such as infants with congenital heart disease (Huisenga et al., 2021), those born prematurely (Luttikhuizen dos Santos et al., 2013), infants exposed to HIV (Siegle & dos Santos Cardoso de Sá, 2018), and children with neurodiverse diagnoses (Torras Mañá et al., 2016).

					Colombia (Rubio-Codina et al., 2016)<br>-See systematic review of cross-cultural validity (Mendonça et al., 2016)	
Capute Scales (Cognitive Adaptive Test/Clinical Linguistic & Auditory Milestone Scale (CAT/CLAMS))<br><br>(Accardo & Capute, 2005)	CAT: visual-motor, problem solving skills. CLAMS: Expressive and receptive language<br><br>Note: CAT/CLAMS classified as an assessment tool, though some mention its use as a screening tool	1 to 36 months	-Cost: $395 USD for Capute Scales Set<br>-Training: those with formal education in healthcare stream<br>-See: www.brookespublishing.com	-Up to 20 minutes to complete<br>-Used by clinicians and pediatricians to screen children with parent report (yes/no) and observations	-Available in several languages such as English, Spanish, and Russian (Eernisse, 2017)	Designed to differentiate between global developmental delay and language problems (Eernisse, 2017).
Child Observation Record (COR) Advantage<br><br>(Foundation, 2013)	Approaches to learning, social & emotional, physical health & development, language, literacy, communication, math, creative arts, science & technology, social studies	Birth to kindergarten	-Cost: ~$70 USD<br>-Training: Online and in-person training with fee based on annual enrollment (certification valid for 3 years after ≥ 80% reliability established with test; see: www.kaymbu.com)	-Lengthier assessment lasting 2-3 months<br>-Teachers (mainly) observe children and provide scores ranging from 0 to 7	-Evidence for validity found only in the US (Wakabayashi et al., 2019)<br>-English and Spanish versions available (www.kaymbu.com)	COR-2 is widely utilized in US Head Start programs (Barghaus & Fantuzzo, 2014).
Comprehensive Developmental Inventory for Infants and Toddlers (CDIIT)<br><br>(Wang, 2003)	Cognition, language, fine motor, gross motor, social, self-help skills	3 to 71 months	-Cost: Unable to locate source for purchase<br>-Training: Requires a trained administrator to perform the CDIIT (e.g., occupational therapists, physical therapists), but primary caregivers report social and self-help subscales (Tsai et al., 2016)	-Unable to identify time required for assessment, but may be lengthier than 30 minutes (screening tool also available)<br>-Includes five subtests and a behaviour rating scale<br>-Every item is scored o (fail) or 1 (pass) by healthcare professionals (e.g., teachers, physical therapists) following training to obtain individual and whole test scores (Tsai et al., 2016)	-Designed with validity and reliability measures in Taiwan (Huang, Tung, Chou, Chou, et al., 2018; Huang, Tung, Chou, Wu, et al., 2018; Hwang, 1987; Tsai et al., 2016)	Validity or reliability measured with full-term and pre-term children (Hwang et al., 2010; Liao et al., 2008) and children with developmental disabilities (Huang, Tung, Chou, Chou, et al., 2018; Huang, Tung, Chou, Wu, et al., 2018) such as Prader Willi and Marfan syndrome (Tsai et al., 2016).
Gesell Developmental Observation Revised (GDO-R)	Cognitive, language, motor, and social-emotional responses in five strands: developmental, letter and numbers, language	2.5 to 9 years	-Cost: $300 USD for complete GDO-R kit<br>-Training: Only trained examiners can administer this test	-Approximately 45 minutes to complete<br>-Requires direct observation of the child to evaluate all domains	-Standardized in the US	Identified children with language delays, visual perception problems, learning disabilities (attention deficit hyperactivity disorder),

(Guddemi et al., 2012)	and comprehension, visual and spatial, social, emotional, adaptive		(https://www.gesell-yale.org/)	-21 tasks are to be examined (https://www.gesell-yale.org/)		and emotional problems (Eck, 2011).
Griffiths Mental Development Scales (GMDS)<br><br>Newest Edition: 3rd Edition<br><br>(Stroud et al., 2016)	3rd Edition: Foundations of learning, language and communication, eye and hand coordination, personal-social-emotional, gross motor skills	Griffiths-II: 0 to 8 years<br>Griffiths-III: 0 to 6 years	-Cost: $1999 USD for entire kit<br>-Training: Doctorate or Master's (with additional training in neuropsychological assessment) required to utilize the tool along with training courses (https://www.aricd.ac.uk/about-the-griffiths-scales/griffiths-iii/to-be-a-new-griffiths-iii-user/)	-Approximately 1 hour assessment<br>-Each of the five scales are assessed by a pediatrician, psychologist, or allied health professional under supervision and weighted equally<br>-Individual scales can also be tested	-GMDS standardized in the United Kingdom and Republic of Ireland (https://www.wpspublish.com/griffiths-iii-griffiths-scales-of-child-development-3rd-edition)<br>-Distributed worldwide such as Australia, South Africa, Israel, US, and Sweden (Cronje, 2021)	GMDS utilized with diverse groups such as typically developing children or those with autism spectrum disorder or Down syndrome (Cronje, 2021).
Mullen Scales of Early Learning (MSEL)<br><br>(Mullen, 1995)	Gross motor, visual reception, fine motor, expressive language, receptive language	0 to 68 months	-Cost: $1168 USD for the complete kit<br>-Training: Requires highly trained professionals (e.g., healthcare degree, master's degree in psychology) to administer the assessment	-Requires approximately 15 minutes (1-year-old)-1 hour (5-year-old) for completion<br>-Materials include: examiner's manual; administration book; stimulus book; protocols; and toys<br>-Scoring completed manually or with computerized software using T-scores	-Normed in US (Bradley - Johnson, 1997)<br>-Reliability and validity widely tested, such as in Africa (Bodeau-Livinec et al., 2019) and rural Guatemala (Colbert et al., 2020)	Used with diverse groups, such as children with autism spectrum disorder (Nordahl-Hansen et al., 2014; Swineford et al., 2015), cerebral palsy and epilepsy (Thomas G Burns et al., 2013), and Rett syndrome (Clarkson et al., 2017).
NEPSY: A Developmental Neuropsychological Assessment (NEPSY)<br><br>Newest Edition: 2nd Edition<br><br>(Korkman et al., 2007)	Executive functioning/attention, language memory/learning, sensorimotor functioning, visuospatial processing, social perception	3 to 16 years of age	-Cost: $1160 USD for entire kit<br>-Training: Requires a high level of expertise in test interpretation (e.g., doctorate degree, licensure, or certification with extensive training or education in that field) (https://www.pearsonassessments.com/)	-Requires 45 minutes to an hour for general assessment of preschool children and school children, respectively, or 90 minutes to 2-3 hours for full assessment<br>-Behavioural observations in clinical settings by trained professional (https://www.pearsonassessments.com/)	-First published in Finnish and then in Swedish (Brooks et al., 2009)<br>-Standardized in Finland and in the United States (Brooks et al., 2009)<br>-Revised and expanded for use in North America (Brooks et al., 2009)<br>-Translated in 8 different languages (Brooks et al., 2009)	Used among different groups in addition to typically developing children (Birch, 2015), including preterm children (O'Meagher et al., 2019), and children with attention deficit hyperactivity, reading, language, autistic, Asperger's, or mathematics disorders, among others. (https://www.pearsonassessments.com/)
Vineland Adaptive	Adaptive functioning domains	Birth to 90 years of age	-Cost: $245 USD for entire kit	-Time to complete varies based on form used and	-Normed in the US	Can be used to measure adaptive behaviour of

Behavior Scales (VABS)<br><br>Newest Edition: 3rd Edition<br><br>(Sparrow et al., 2016)	(communication, daily living skills, and socialization), motor skills, maladaptive behaviours		-Training: Requires Master's degree in related degree (e.g., psychology) or certification or degree related to this field; training required via online or in-person courses (https://www.pearsonassessments.com/)	whether motor skills and maladaptive behaviours are assessed: interview format requires 20 to 60 minutes, parent/caregiver self-report requires 30 to 60 minutes, and teacher form is 8 to 15 minutes<br>-For those in the range from birth to 90 years of age, requires an interview and a parent/caregiver form to score; those within the range of 3 to 21 years of age also require a teacher form<br>-Scored via Q-global or manual scoring (https://www.pearsonassessments.com/)	-Translation provided in Spanish	individuals with intellectual and developmental disabilities, autism spectrum disorder, attention deficit hyperactivity disorder, post-traumatic brain injuries, and hearing impairments (note age groups are unclear) (https://www.pearsonassessments.com/).

Table S2.

Reliability and validity of screening and assessment tools

Tool Name	Reliability			Validity		
	**Internal Consistency Reliability**	**Intra-rater**	**Inter-rater**	**Structural, discriminant, and discriminative**	**Concurrent (i.e., criterion) and Convergent (i.e., construct)**	**Predictive (i.e., criterion)**
Ages and Stages Questionnaire (ASQ)	-ASQ-3 Cronbach alphas: .51- .87 in user guide (J. Squires et al., 2009)<br>-Lower for 2-2.5 year translated & adapted versions (Velikonja et al., 2017)<br>-Cronbach's alpha = 0.719 for gross motor (Yue et al., 2019a)<br>-Cronbach's alpha ranged from 0.77 to 0.88 in Mexican sample (Ortiz-León et al., 2018)	-92% over two weeks (J. Squires et al., 2009)<br>-ICC of 0.90 for personal-social and 0.99 for gross motor after 2 weeks in an Indigenous Australian sample (Simpson et al., 2021)<br>-High agreement for parents after 2 weeks (Zirakashvili et al., 2018)<br>-High Pearson correlations (0.80 to 0.91) after two weeks in Peruvian sample (Chong et al., 2017)	-93% agreement between parents and trained test examiners ICC = .43-.69 (J. Squires et al., 2009)<br>-88% agreement between raters (Simpson et al., 2021)<br>-Moderate to high correlation between parents and teachers (0.43 to 0.85) except for 42 months of age (Ortiz-León et al., 2018)<br>-High agreement (r=0.93) between parents and child educators for fine motor (Lopes et al., 2015)	-16-month measured child development with 5 domains; however, problem solving highly correlated (r=.94) with fine motor (Chen et al., 2018)<br>-Differing age ASQs measure 5 factors (Olvera Astivia et al., 2017)<br>-Discriminant validity based on gestational age, birth weight, and developmentally delayed children (Shrestha et al., 2019)	-Validity between ASQ-3 and developmental tools (e.g., Bayley-II, BDI-II, PEDS) (Singh et al., 2017)<br>-Based on a review of 43 studies, sensitivity (.77) and specificity (.81) to diagnose development delay (Muthusamy et al., 2022)<br>-ASQ-3 overall and age-specific sensitivities greater than 70% with the BSID-III (Yunilda et al., 2023)	-Widely tested (e.g., preschool ASQ-3 scores predicted school age *(Schonhaut et al., 2020)*<br>*-Predicted cognitive and school performance (Martínez-Nadal et al., 2021)*<br>*-Predicted gross motor difficulties in young children (Fauls et al., 2020)*
Assessment, Evaluation, and Programming System for Infants and Children (AEPS)	Cronbach's alpha = .825 (gross motor in typically developing children) to .985 (cognitive of total group) with AEPS-2 (Noh, 2005)	Test-retest (e.g., *r*s = .77-.96) established for first edition (Winchell, 2011)	-AEPS-2 inter-rater for children with (.81) and without disabilities (.72) (Noh, 2005)<br>-80% trainer and teacher (Gao, 2008)<br>-89% between teacher/provider and AEPS-3 expert (Grisham et al., 2021)	-6 factors (Winchell, 2011) -AEPS-1 and -2: lower scores for children with disabilities (Hsia, 1993; Noh, 2005)	-AEPS-2, AEPS-3 correlated with BDI-2 (e.g., AEPS-2 social communication with BDI-2 expressive and receptive communication, *r* = .63-.76) (Gao, 2008; Grisham et al., 2021)	No evidence found but did/could not access manual.
Australian Developmental Screening Test (ADST)	No evidence found but did/could not access manual.	No evidence found but did/could not access manual.	No evidence found but did/could not access manual.	No evidence found but did/could not access manual.	Sensitivity (95%), specificity (52%) with GMDS, specificity improved with modified criteria (McDonald & Milne, 2013; Morris et al., 2012)	No evidence found but did/could not access manual.
Battelle Developmental Inventory (BDI) (includes screening test)	-BDI-II Cronbach's alpha from .79 (cognitive) to .89 (communication) (Rubio-Codina et al., 2016)	-BDI-II test-retest ICC values from .71 (personal-social) to .98 (motor) (Rubio-Codina et al., 2016)	-Coefficients ≥ .85 (Snyder et al., 1993)<br>-.97 for infants with known or suspected disabilities (Boyd et al., 1989)	-5 factors established in BDI-II Examiner's Manual<br>-Rasch analysis confirmed gross motor structural validity of	-BDI-II validity with numerous tests (e.g., Bayley III, DDST-II, MSEL) (Frisk et al., 2009; Moyal, 2010; Rubio-Codina et al., 2016)	-BDI Screening for children with Fragile X Syndrome: developmental delay sensitivity at 9 months with MSEL = 54% for boys, 75% for girls; at 18 months = 83% for

	-Average Cronbach's alpha = .906 for BDI-II (Ma, 2012)<br>-High individual domain scores with value of .93 through Rasch modeling (Elbaum et al., 2010)	-Coefficient of .80 after six weeks (Boyd et al., 1989)<br>-.80 as reported by Goldin et al. (2014), captured in a review by (Cunha et al., 2018)	-Agreement between two examiners = 95% (McLean et al., 1987)	BDI-II Screening test (LaForte, 2014)<br>-Rasch analysis confirmed validity (i.e., 80% of correlations more than .5)(Elbaum et al., 2010)		boys, 50% for girls (Mirrett et al., 2004)<br>-BDI-II .94 sensitivity and .31 specificity identifying children with autism (*SD* -1.5) (Sipes et al., 2011)<br>-Strongly predicted academic achievement (Guidubaldi & Perry, 1984)
Bayley Infant Neurodevelopmental Screener (BINS)	-Cronbach's alpha = .73 to .85 based on test manual by Aylward (1995), as cited by (Johnson, 1997)<br>-Cronbach's alpha = .67-.76 in Brazil (Guedes et al., 2011)<br>-.77- .86 in South Africa (Rodriguez et al., 2020)	-Test-retest after 1 to 2 weeks in South America = .80-.93 (McCarthy et al., 2012)<br>-Test-retest ranged from .71 to .84 (3 to 18 months) based on test manual by Aylward (1995), as cited by (Johnson, 1997)	-84% physician inter-rater agreement with training videos (McCarthy et al., 2012)	-BINS Rasch model map (Guedes et al., 2011)<br>-5-factor structure observed in rural South Africa (Rodriguez et al., 2020)	-BINS correlated with neurological assessments (.35-.36), DDST-II (.59-.62), BSID-II (.36-.62)(Guedes et al., 2011) and parent reports (.65-.85)(Aylward & Verhulst, 2008)	-BINS in infancy predicted BSID-II (Hess et al., 2004; Soysal et al., 2014)<br>-67% to 76% validity for identifying at-risk infants (Leonard et al., 2001)
Bayley Scales of Infant and Toddler Development (BSID)	-BSID-3 Cronbach's alpha = .97 in US (L'Hotta et al., 2020), .83-.9 for adapted subtests in Vietnam (Nguyen, 2017), and .88-.96 for version in Kenya (McHenry et al., 2021)	-Average BSID-3 stability coefficients ≥ .80 (Albers & Grieve, 2007)<br>-Test-retest reliability in Taiwan: ICC = .85-.99 (Yu et al., 2013)<br>-Systematic review on BSID-3 motor reliability (Spittle et al., 2008)	-Inter-rater ICC > .90 for BSID-3 adapted in Ethiopia (Hanlon et al., 2016) and >.95 in Nepal (Ranjitkar et al., 2018)<br>-Moderate to excellent inter-rater reliability = .76-.97 in Taiwan (Lin et al., 2020)	-Rasch analysis for children with sickle-cell anemia (L'Hotta et al., 2020)<br>-BSID-3 structural validity assessed internationally (e.g., Peru, India, Nepal; Pendergast et al., 2018), and Kenya (McHenry et al., 2021)	-BSID used as gold standard (Albuquerque et al., 2018; Siegle & dos Santos Cardoso de Sá, 2018; Yue et al., 2019b)	-BSID predictive validity (e.g., intellectual, language outcomes) in children born preterm (Månsson et al., 2019), with autism (Torras Mañá et al., 2016), and without disabilities (Krogh & Væver, 2019)
BRIGANCE Screens III (BRIGANCE)	-Internal consistency reliability (total scores = .94-.99) for BRIGANCE-III (French, 2013)	-BRIGANCE-III test-retest (*M* = 12 days) total scores = .92-.99 (French, 2013)	-BRIGANCE-III inter-rater reliability .93 -.96 (French, 2013)	-Confirmatory factor analysis supports BRIGANCE-III structure (French, 2013)	-BRIGANCE-III correlated with BDI-2, MSEL, BSID-3; Children with disabilities scored lower than children without (French, 2013)<br>-BRIGANCE-II 100% sensitivity and 35-60% specificity with BSID-3 (Dempsey et al., 2016)<br>-Correlation (r=0.67) between BRIGANCE preschool screen and ASQ communication (Frisk et al., 2009)	-No evidence found but did/could not access manual.

Capute Scales (Cognitive Adaptive Test/Clinical Linguistic & Auditory Milestone Scale (CAT/CLAMS))	No evidence found but did/could not access manual.	No evidence found but did/could not access manual.	No evidence found but did/could not access manual.	No evidence found but did/could not access manual.	-CAT/CLAMS validity assessed with BSID-3 for developmental and intelligence measures (Larson, 2016)<br>-Strong correlations with BSID (Wang et al., 2005)<br>-Correlated strongly with the BSID with coefficients $\geq$ .63 (Wachtel et al., 1994)	-Predicted significant developmental delay at 2 years of age (Wang et al., 2005)<br>-High predictive validity of development concerns 6 and 12 months later (Wachtel et al., 1994)<br>-High predictive validity with BSID mental development index and within test 12 months later (Rossman et al., 1994)
Child Development Inventory (CDI)	-Cronbach's alpha = .33 (5 year old gross motor) to .96 (2 year old expressive language) (Ireton, 1992)<br>-Cronbach's alpha ranging from .77 to .92 and from .70 to .94 for 13 to 18 month and 19 to 24 month Chinese infants, respectively (Wu, 1997)	-French CDI test-retest = .97 (Duyme et al., 2011)	-French CDI inter-rater = .76 between parents and teachers (Duyme et al., 2011)	-Score increase with age indicates developmental progress: *r*s from .70 (letters) to .89 (general development) (Ireton, 1992); similar findings with French CDI (Duyme et al., 2011)	-Correlated with BDI, Preschool Language Scale-3 (Musser, 2001), and BSID-2 (Doig et al., 1999)<br>-73-100% sensitivity and 87-97% specificity with CAT/CLAMS (Doig et al., 1999; Montgomery et al., 1999)	-CDI general development Kindergarten outset highest predictor of reading (r = .69) and math (r = .59) at year end (Ireton, 1992)
Child Development Review (CDR)	No evidence found but did/could not access manual.	No evidence found but did/could not access manual.	No evidence found but did/could not access manual.	No evidence found but did/could not access manual.	-Sensitivity (.44) and specificity (.80) with BSID-3 (Dempsey et al., 2016)<br>-Social, fine motor, and language significantly correlated (.28-.54) with BSID-2 (Voigt et al., 2007)	No evidence found but did/could not access manual.
Child Observation Record (COR) Advantage	-Cronbach's alpha for older version Preschool COR = .89-.96 (Li, 2016)<br>-Internal consistency reliability = 0.86 to 0.95 (Fantuzzo et al., 2002)	No evidence found for test-retest reliability based on this review.	-Inter-rater average agreement = 86% (78% for approaches to learning to 94% for social studies) (Wakabayashi et al., 2019)	-COR Advantage difficulty increased with age (Wakabayashi et al., 2019)<br>-Confirmatory factor analysis for Preschool COR-2 identified structure concerns (Barghaus & Fantuzzo, 2014)<br>-Evidence for divergent validity (Sekino & Fantuzzo, 2005)	-Validity of COR Advantage with BSID-3 (*r*s =.74 - .90) and achievement tests (Wakabayashi et al., 2019)<br>-Evidence for convergent validity (Fantuzzo et al., 2002; Sekino & Fantuzzo, 2005)	No evidence found for predictive validity based on this review.
Comprehensive Developmental Inventory for Infants	-Standardized in Chinese; Cronbach's alpha .75-.99 (Hang et	-Whole test ICC for preterm (.95) and term infants (.93), and	-Whole test ICC for preterm infants (.98), term infants (1.00), and	-2 factor in infant and 1-year-olds and 3 factors in 2-year-olds and	-Developmental quotient correlated (r = .57-.67) with BSID-2 (Liao et al., 2008)	No evidence found but did/could not access manual.

and Toddlers (CDIIT)	al., 1998 cited in Hwang et al., 2010)	children with disabilities (.99) (Liao & Pan, 2005)	children with disabilities (.97) (Liao & Pan, 2005)	preschool groups (Hwang et al., 2010)		
Copenhagen Infant Mental Health Screening (CIMHS)	No evidence found but did/could not access manual.	No evidence found but did/could not access manual.	No evidence found but did/could not access manual.	-Confirmatory factor analysis found 5 factors (Ammitzbøll et al., 2016)	No evidence found but did/could not access manual.	-3+ problems on 9-to-10-month screening: 74% sensitivity, 50% specificity to detect mental health disorders at 1.5 years (Ammitzbøll et al., 2018)
Denver Developmental Screening Test (DDST)	-Cronbach's alpha .97 for Portuguese version (Boo et al., 2020) and .9-.93 in Bogota (Rubio-Codina et al., 2016)<br>-Kuder-Richardson Index = .98 for Spanish version full scale (De-Andrés-Beltrán et al., 2015)	-Test-retest .49 (personal-social) to.93 (language) (Rubio-Codina et al., 2016)<br>-98% intra-rater agreement (De-Andrés-Beltrán et al., 2015)<br>-ICC of .90 (fine motor-adaptive) to .95 (personal-social) in Tehran (Shahshahani et al., 2010)	-97% inter-rater reliability with Portuguese version (Lopez Boo et al., 2020), .96 in Sri Lanka (Wijedasa, 2012), and 96% for Spanish version (De-Andrés-Beltrán et al., 2015)	-Good structure fit with Portuguese version (Lopez Boo et al., 2020)	-DDST-II correlated with BSID-3 (Rubio-Codina et al., 2016) and GMDS (Luiz et al., 2004)<br>-Sensitivity (.89) and specificity (.92) when identifying developmental delay (De-Andrés-Beltrán et al., 2015)	-DDST-II predictive validity of intelligence increased with age (Rubio-Codina et al., 2020)<br>-DDST-II predicted MDI of the BSID (Pederson et al., 1988)
Developmental Assessment of Young Children (DAYC)	-.89-.98 across 5 domains (Swartzmiller, 2014)<br>-Kuder-Richardson values .89-.99 for Arabic version across the five domains (Rawan M Abu Saleh & Jamil M Smadi, 2017)	-1-2 week test-retest = .70-.91 (Swartzmiller, 2014) and 2 week = .996 (Rawan M. Abu Saleh & Jamil M. Smadi, 2017)	No evidence found for inter-rater reliability based on this review.	-Lower scores for children with disabilities (Rawan M Abu Saleh & Jamil M Smadi, 2017)<br>-Increasing scores with age, lower scores for children with disabilities (Swartzmiller, 2014)	-Correlated with BDI-2 (Swartzmiller, 2014)	-Predictive tool for cerebral palsy alongside imaging (Novak et al., 2017)
Developmental Indicators for the Assessment of Learning (DIAL)	-DIAL-4 reliability= .80 and .90 for English & Spanish (Coughlan, 2015)<br>-Speed DIAL-4 =.84-.94 for English version (Doskey et al., 2013)<br>-Estimates for 6-month age group ranged from .71 to .93 for Spanish version (Garcia de Alba, 2007)	Test-retest=.64 (motor, English) =.95 (language, Spanish) (Coughlan, 2015)<br>-Speed DIAL-4=.78-.84 (English) and .89 (Spanish) (Doskey et al., 2013)<br>-Correlation coefficients ranged from .97 (motor) to .99 (total score) 2 weeks later with Taiwanese adaptation (Mardell-Czudnowski et al., 1986)	-Average inter-rater agreement = .89-.98 (Coughlan, 2015)<br>-Percent agreement values above .81 for motor, concept, and communications subtest among 16 DIAL scorers (Mardell & Goldenberg, 1976)	-Correlations between subtests of Speed DIAL and DIAL-4 establish construct validity of Speed DIAL (Doskey et al., 2013)<br>-Describes performance well in diverse demographic and minority groups (Assel & Anthony, 2009)<br>-3-factor structure is appropriate for English and Spanish samples (Garcia de Alba, 2007)	-DIAL-4 correlated with ESP & cognitive test (Coughlan, 2015)<br>-Speed DIAL-4 correlated with DIAL-4, BDI-2, and cognitive measure (Doskey et al., 2013)<br>-Correlation with similar scales of the Learning Accomplishment Profile (Barnett et al., 1988)	-DIAL-3 (e.g., concepts, language) pre-Kindergarten predicted literacy and reading years later (Katz, 2016)<br>-DIAL total score predicted achievement on standardized tests and student achievement from teacher ratings (Spagnola, 2009)<br>-DIAL communications subtest most valid single predictor of school success; DIAL Concepts subtest was the only significant predictor of Progress Report composite score (Obrzut et al., 1981)

Developmental Profile (DP)	-DP-4 internal consistency reliability: .80-.93 for 0-5 years (Alpern, 2020)	-DP-4 test-retest general development (not limited to 0-5 years) = .65-.84 (Alpern, 2020)	-DP-4 parent checklist inter-rater for 0.5-12 years (e.g., 2 parents) = .73-.86 (Alpern, 2020)	-Confirmatory factor analysis found items loaded onto general development (Alpern, 2020)	-Convergent validity with developmental tools (e.g., DP-3, DAYC-2) (Alpern, 2020)	-Predicted scores on DDST (Burgess et al., 1984)
Early Screening Inventory – Revised (ESI-R)	No evidence found but did/could not access manual.	.76 - .84 test-retest reliability (Meisels et al., 1993)	-ESI inter-rater reliability >.98 (Meisels et al., 1993)	-ESI discriminated between rescreened and referred (lower scores) groups and children scored more items with increasing age (Meisels et al., 1993)	No evidence found for convergent or concurrent validity based on this review.	-ESI-R predicted McCarthy Scales for older children (e.g., 5 years) with correlation of .83 and sensitivity and specificity values above .80 (Meisels et al., 1993)
Early Screening Profiles (ESP)	-Based on test review of manual, moderate to high alpha coefficients except for motor scale (Snow, 1995)	-Moderate to high correlations within 21 days of retesting for motor profile (.70), self-help (.81, and cognitive profile (.90); lower correlations after 21 days (Smith, 1990)<br>-High retest within 21 days for language scale (.91) and between 22 to 75 days for language scale (.88) based on test review of manual (Snow, 1995)<br>-Ranged from .70 to .93 after average of 10 months (Smith, 1991)	No evidence found but did/could not access manual.	-In manual, correlations between subtests are higher than domains as reported in a test review (Snow, 1995)	-High correlation with the Differential Ability Scales ($r \geq .43$) (McIntosh et al., 2000)<br>-High correlation with the Kaufman Assessment Battery for Children, particularly between ESP global scales and K-ABC achievement (Lasee & Smith, 1991)<br>-Correlated well with the Stanford Binet (SB) except for the cognitive scale of the ESP with the quantitative reasoning of the SB (Genteman, 1992)	-Based on test review of manual, predictive validity present (Snow, 1995)
Gesell Developmental Observation-Revised (GDO-R)	No evidence found for internal consistency reliability based on this review.	No evidence found for intra-rater reliability based on this review.	-Based on technical report, inter-rater values of .91-.93 (Guddemi et al., 2012)	-Some evidence for construct validity (Guddemi et al., 2012)	No evidence found for convergent or concurrent validity based on this review.	No evidence found for predictive validity based on this review.
Griffiths Mental Development Scales (GMDS)	-Cronbach's alpha for some of the Chinese version GMDS subscales (e.g., locomotor, personal-social) >.70 (Tso et al., 2018)	-GMDS-III test-retest reliability = .967-.996 (Cronje, 2021)	-Inter-rater reliability = .967-.996 in United Kingdom and Ireland (Cronje et al., 2022)<br>-High inter-rater agreement between two (.9855) and three (.8525) testers in agro-industrial Philippine province (Reyes et al., 2010)	-Factor analysis in South Africa found some differences noted between ethnicities (Luiz et al., 2001)	-GMDS-III correlated with developmental (e.g., ASQ-3, intelligence measures)(Cronje, 2021)<br>-GMDS-II correlated with BSID-II (r=.82-.86) for high risk (e.g., born preterm) children in Switzerland (Cirelli et al., 2015)<br>-Almost all correlations (41/45) between subtests of GMDS and Battelle above .70 (Venter & Bham, 2003)	-Used for infants born prematurely to predict school age cognitive deficits (Wong et al., 2016)<br>-Some scales predicted IQ scores on the Wechsler preschool and primary scale of intelligence (Sutcliffe et al., 2010)<br>-Moderate to high predictive validity for IQ and DQ later in life (Larg et al., 1990)

Infant Developmental Inventory (IDI)	No evidence found for internal consistency reliability based on this review.	No evidence found for intra-rater reliability based on this review.	No evidence found for inter-rater reliability based on this review.	No evidence found for structural, discriminant, or discriminative validity based on this review.	No evidence found for convergent or concurrent validity based on this review.	No evidence found for predictive validity based on this review.
Infant-Toddler Checklist (ITC)	-Internal consistency reliability of .70-.84 in Sweden when excluding speech (Faldt et al., 2021) and .75 for social and .82 for total (Eadie et al., 2010)<br>-High Cronbach's value for total raw score (.96) (Hamrick et al., 2020)	-Test-retest total raw score *r*=.87 and total standard *r*=.84 in US (Wetherby et al., 2002)<br>-Test-retest in with Chinese version=.62-.77 (Lin & Chiu, 2014)	-Inter-rater reliability between mothers and fathers in Croatia; ICC=.87-.94 (Cepanec et al., 2012)<br>-Inter-rater of .78 to .84 (Lin & Chiu, 2014)	-Satisfactory three factor model for Swedish version (Fäldt et al., 2021) and Australian sample (Eadie et al., 2010)<br>-Evidence for differentiation across age groups and different clinical groups (Hamrick & Tonnsen, 2019)	-ITC correlated with receptive (*r* = .38-.66) and expressive language at 2 years (Wetherby et al., 2002)	- ITC at 18 months associated with developmental diagnosis at 3-5 years (Borkhoff et al., 2022)<br>-ITC in infancy predicted autism spectrum disorder and developmental delays at 36 months; sensitivity =51-62% and specificity= 42-85% (Parikh et al., 2021)<br>-ITC at 12-16 months and 17-21 months predicted language at 2 years (Wetherby et al., 2002)
Mullen Scales of Early Learning (MSEL)	MSEL internal consistency reliability for five scales, 80% were less than .85 (Bradley-Johnson, 1997)<br>-Adapted, translated MSEL version in Guatemala Cronbach's alpha = .91-.93 (Colbert et al., 2020)<br>-Cronbach's alpha values ranged from .84 to .93 for Taiwanese version (Cheong et al., 2022)	-MSEL test-retest ranged from .70s (22-56 months) to .96 (1-24 months) (Bradley-Johnson, 1997)<br>-Guatemalan -MSEL test-retest via *t*-test comparisons over 1 year: 0.83-1.06 (Colbert et al., 2020)<br>-Test-retest (measured with ICC) above .95 (Eigsti et al., 2010)	-MSEL inter-rater = .91-.99 (Bradley-Johnson, 1997)<br>-Inter-rater reliability for Guatemalan version of MSEL ICC = .99-1.0 (psychologists, neuropsychologists) (Colbert et al., 2020)<br>-High inter-rater agreement with ICCs of .96 to .99 in Taiwanese version (Cheong et al., 2022)<br>-High inter-rater reliability between nurses and gold standard interviewer (>94%) (Koura et al., 2013)	-Confirmatory factor analyses conducted for children with and without autism spectrum disorder (Swineford et al., 2015)<br>-Children with cerebral palsy, ASD, and epilepsy attained lower MSEL scores than children from a normative sample (Thomas G. Burns et al., 2013)<br>-Ability to differentiate between typically developing children and those with global development delay or autism spectrum disorder (Cheong et al., 2022)	-MSEL language correlated with language scales of the CDI (.81-.90) in children with autism spectrum disorder (Nordahl-Hansen et al., 2014)<br>-MSEL correlated (90-.91) with cognitive and achievement test (Farmer et al., 2016)<br>-Adapted MSEL correlated with adaptive measure in children with Rhett syndrome (Clarkson et al., 2017)	-Early learning composite at 2 years predicted (*r*=.46) intelligence at age 6 (Girault et al., 2018)<br>-Visual reception predicted IQ score for children with deafness (Caudle et al., 2014)<br>-Receptive language at 20 months predicted speech impairment at 4 (Bishop et al., 2012)
NEPSY: A Developmental Neuropsychological Assessment (NEPSY)	-Adequate to high internal consistency reliability based on test review (Davis & Matthews, 2010)	-High test-retest correlation values for some subtests such as .91 for picture puzzles after mean of 21 days, based on test review and for older ages (Davis & Matthews, 2010)	-High inter-rater reliability of 93 to 99% based on test review (Davis & Matthews, 2010)	-Rasch modeling supported the capacity to use NEPSY-II to measure affect recognition ability in young children (Yao et al., 2018)<br>-Correlations with measures of verbal ability and executive	-Zero-order correlations were significant with other measures (Tuerk et al., 2021)<br>-Sufficient evidence of concurrent validity with several tools based on test review (Davis & Matthews, 2010)<br>-NEPSY-II subdomains correlated with parent-	-NEPSY-II at 4 years predicted some executive functioning scores one year later (O'Meagher et al., 2019)<br>-NEPSY-II Statue subtest at 4 years predicted children with attention deficit hyperactivity

				functioning (Annotti & Teglasi, 2017)<br>-NEPSY distinguished between typically developing children and those with hyperactivity and inattention (Rajendran et al., 2015)	reported executive function measure for children with perinatal arterial ischemic stroke (Krivitzky et al., 2019)	disorder at age 6 (Breaux et al., 2016)
Nipissing District Development Screen (NDDS); Now Looksee Checklist	No evidence found for internal consistency reliability based on this review.	No evidence found for intra-rater reliability based on this review.	No evidence found for inter-rater reliability based on this review.	No evidence found for structural, discriminant, or discriminative validity based on this review.	-Moderate agreement with the ITC with Cohen κ = 0.45 (van den Heuvel et al., 2016)	No evidence found for predictive validity based on this review.
Parents' Evaluation of Developmental Status (PEDS)	-Dutch translations Cronbach's alpha = .70 (parents) and .60 (professional caregivers) (Doove et al., 2019)	-Test-retest ICC for Dutch translation = .80 (Doove et al., 2019) and .812 for Mandarin translation (Toh et al., 2017)	-High inter-rater reliability between author and trained nurse = .87 (Toh et al., 2017)<br>-Percent agreement between teachers and parents ranged from 73% (behavior) to 80% (social-emotional) (Wake et al., 2005)	No evidence found but did/could not access manual.	-Agreement between online format and paper tool (Maleka et al., 2016)<br>-Moderate to high sensitivity (78.9%) for severe delays and specificity (79.6%) for no delays among 0-42 months, using BSID and BDI as reference (Sheldrick et al., 2020)	-PEDS concerns (e.g., self help, motor) predicted lower academic scores (e.g., reading) 2 years later (Wake et al., 2005)<br>-PEDS identified 85.9% of children at risk of developmental delay via online format (du Toit et al., 2021)<br>-PEDS sensitivity (26-94%) and specificity (64-91%) in Dutch sample (Doove et al., 2019)
Parents' Evaluation of Developmental Status Developmental Milestones (PEDS DM)	-Guttman's λ coefficient = .98 (Kyle B Brothers et al., 2008)<br>-Cronbach's alpha =.89 for total score on Jordanian version (Mattar & Arouri, 2017)	-Test-retest reliability = .98-.99 (Kyle B Brothers et al., 2008) and .88-.92 for Jordanian version (Mattar & Arouri, 2017)	-Inter-rater reliability =.82-.96 (Kyle B Brothers et al., 2008)	-Correlations between items and subscales on Jordanian version: .303-.725 fine motor, .218-.545 self-help, .202-.726 receptive language, .384-.624 expressive language, .254-.729 gross motor, .236-.623 social emotional (Mattar & Arouri, 2017)	-Sensitivity ≤ 16th percentile on diagnostic tools = 83% and specificity = 84% (Kyle B. Brothers et al., 2008)<br>- Jordanian version correlated (.79 to .84) with the Preschool and Kindergarten Children's Performance scale (Mattar & Arouri, 2017)	No evidence found for predictive validity based on this review.
Preschool Developmental Assessment Scale (PDAS)	Kuder Richardson 20 was .93 for cognitive (Leung et al., 2013) and language (Wong et al., 2012) and .86 for social (Leung et al., 2011); note: these papers are considered only one publication as they only examined one domain each time (creating a test manual)	Cognitive = .81 (Leung et al., 2013) and language = .91 (Wong et al., 2012).	No evidence found for inter-rater reliability based on this review.	Rasch analyses for social, language, and cognitive (Leung et al., 2011; Leung et al., 2013; Wong et al., 2012).	Cognitive, language, and social correlated with intelligence, language (Leung et al., 2013; Wong et al., 2012).	No evidence found for predictive validity based on this review.

Revised Denver Prescreening Developmental Questionnaire (R-PDQ)	-Cronbach's alpha > .80 in Tehran (Shahshahani et al., 2011)	-94% 1-week test-retest reliability (Frankenburg et al., 1987)	-Inter-rater kappa = .89 in Tehran (Shahshahani et al., 2011)<br>-Inter-rater (teachers, parents) = 83% (Frankenburg et al., 1987)	No evidence found but did/could not access manual.	-R-PDQ identified 84% nonnormal DDST results (Frankenburg et al., 1987)<br>-PDQ and DDST had 93% mean agreement with families with low incomes (Rosenbaum et al., 1983)<br>-PDQ and DDST had agreement scores above 90% at 3, 6, and 9 months (Rosenbaum, 1981)	No evidence found for predictive validity based on this review.
Shoklo Developmental Test (SDT)	No evidence found for internal consistency reliability based on this review.	No evidence found for intra-rater reliability based on this review.	Perfect agreement for 11/15 (73%) infants (Haataja et al., 2002).	No evidence found for structural, discriminant, or discriminative validity based on this review.	-Total correlated (*r* = .74) with Griffiths Developmental Quotient (Haataja et al., 2002)	No evidence found for predictive validity based on this review.
The Toddler Language and Motor Questionnaire (TLMQ)	-Most Cronbach's alpha values (54/70) were > .80 (Gudmundsson, 2015)	-Cited from Icelandic publication: .79-.89 language (Gudmundsson, 2015)	No evidence found for inter-rater reliability based on this review.	-2 factors (motor, language) (Gudmundsson, 2015)	-Correlated with measures (e.g., intelligence, language) (Gudmundsson, 2015)	No evidence found for predictive validity based on this review.
Vineland Adaptive Behavior Scales (VABS)	-Internal consistency reliability of domains greater than 0.90 for VABS Dutch screener (van Duijn et al., 2009)<br>-Vietnamese VABS .76 to .95 except for receptive domain (Goldberg et al., 2009)<br>-High internal consistency reliability (>.90) (de Bildt et al., 2005)	-Pearson correlation ≥ .8 in a Hindi-translated version after 7-10 days (Kumar et al., 2016)<br>-Test-retest reliability values above .90 (van Duijn et al., 2009)	-Pearson correlation ≥ .8 in a Hindi-translated version (Kumar et al., 2016)	-Based on a review (James et al., 2014), evidence for construct validity<br>-Factor analysis used with adapted Dutch version for parents found one factor (Van Duijn et al., 2010)<br>- Vietnamese VABS could discriminate children with and without intellectual disability (Goldberg et al., 2009)	-Concordance correlation > .70 for VABS-2 and VABS-3 (Farmer et al., 2020)<br>- VABS (1 and 2) adaptive composite correlated with MSEL composite (r = .44-.61) for children with cochlear implants (Caudle et al., 2014)<br>- Vineland-2 and Bayley-3 correlated (.61-.82) (Scattone et al., 2011)	-Predicted verbal IQ 17 years later among children with autism spectrum disorder (Anderson et al., 2014)

References

Accardo, P. J., & Capute, A. J. (2005). *The Capute Scales: Cognitive Adaptive Test/Clinical Linguistic & Auditory Milestone Scale (CAT/CLAMS)*. Brookes.

Albers, C. A., & Grieve, A. J. (2007). Test review: Bayley, n. (2006). Bayley Scales of Infant and Toddler Development– third edition. San Antonio, TX: Harcourt assessment. *J Psychoeduc Assess*, *25*(2), 180-190. https://doi.org/10.1177/0734282906297199

Albuquerque, P. L. d., Guerra, M. Q. d. F., Lima, M. d. C., & Eickmann, S. H. (2018). Concurrent validity of the Alberta Infant Motor Scale to detect delayed gross motor development in preterm infants: A comparative study with the Bayley III. *Dev Neurorehabil, 21*(6), 408-414. https://doi.org/10.1080/17518423.2017.1323974

Alfonso, V. C., Rentz, E. A., & Chung, S. (2010). Review of the Battelle Developmental Inventory, second edition. *J Early Child Infant Psychol, 6*, 21-40.

Alpern, G. D. (2020). *Developmental Profile 4: Test manual*. Western Psychological Services

Ammitzbøll, J., Holstein, B. E., Wilms, L., Andersen, A., & Skovgaard, A. M. (2016). A new measure for infant mental health screening: Development and initial validation. *BMC Pediatr*, *16*(1), 197. https://doi.org/10.1186/s12887-016-0744-1

Ammitzbøll, J., Thygesen, L. C., Holstein, B. E., Andersen, A., & Skovgaard, A. M. (2018). Predictive validity of a service-setting-based measure to identify infancy mental health problems: A population-based cohort study. *Eur Child Adolesc Psychiatry, 27*(6), 711-723. https://doi.org/10.1007/s00787-017-1069-9

Anderson, D. K., Liang, J. W., & Lord, C. (2014). Predicting young adult outcome among more and less cognitively able individuals with autism spectrum disorders. *J Child Psychol Psychiatry, 55*(5), 485-494. https://doi.org/https://doi.org/10.1111/jcpp.12178

Annotti, L. A., & Teglasi, H. (2017). Functioning in the real world: Using storytelling to improve validity in the assessment of executive functions. *J Pers Assess, 99*(3), 254-264. https://doi.org/https://doi.org/10.1080/00223891.2016.1205075

Assel, M. A., & Anthony, J. L. (2009). Factor structure of the DIAL-3: A test of the theory-driven conceptualization versus an empirically driven conceptualization in a nationally representative sample. *J Psychoeduc Assess, 27*(2), 113-124. https://doi.org/https://doi.org/10.1177/0734282908324038

Aylward, G. (1995). *Bayley Infant Neurodevelopmental Screener*. San Antonio, TX: The Psychological Corporation.

Aylward, G. P., & Verhulst, S. J. (2008). Comparison of caretaker report and hands-on neurodevelopmental screening in high-risk infants. *Dev Neuropsychol, 33*(2), 124-136. https://doi.org/10.1080/87565640701884220

Barghaus, K. M., & Fantuzzo, J. W. (2014). Validation of the preschool Child Observation Record: Does it pass the test for use in Head Start? *Early Educ Dev, 25*(8), 1118-1141. https://doi.org/10.1080/10409289.2014.904646

Barnett, D. W., Faust, J., & Sarmir, M. A. (1988). A validity study of two preschool screening instruments: The LAP-D and DIAL-R. *Contemp educ psychol*, *13*(1), 26-32. https://doi.org/https://doi.org/10.1016/0361-476X(88)90003-3

Bayley, N., & Aylward, G. (2019). Bayley Scales of Infant and Toddler Development fourth edition (Bayley-4). *Bloomington, MN: NCS Pearson*.

Birch, S. E. (2015). *The relationship between a norm referenced measure of theory of mind and preschoolers' social skills in the classroom*. City University of New York. https://academicworks.cuny.edu/gc_etds/863

Bishop, D. V. M., Holt, G., Line, E., McDonald, D., McDonald, S., & Watt, H. (2012). Parental phonological memory contributes to prediction of outcome of late talkers from 20 months to 4 years: A longitudinal study of precursors of specific language impairment. *J Neurodev Disord*, *4*(1), 1-12. https://doi.org/10.1186/1866-1955-4-3

Bodeau-Livinec, F., Davidson, L. L., Zoumenou, R., Massougbodji, A., Cot, M., & Boivin, M. J. (2019). Neurocognitive testing in west African children 3–6 years of age: Challenges and implications for data analyses. *Brain res bull*, *145*, 129-135. https://doi.org/https://doi.org/10.1016/j.brainresbull.2018.04.001

Boo, F. L., Mateus, M. C., & Sabatés, A. L. (2020). Initial psychometric properties of the Denver II in a sample from northeast Brazil. *Infant Behav Dev*, *58*, 101391. https://doi.org/https://doi.org/10.1016/j.infbeh.2019.101391

Borkhoff, C. M., Atalla, M., Bayoumi, I., Birken, C. S., Maguire, J. L., & Parkin, P. C. (2022). Predictive validity of the Infant Toddler Checklist in primary care at the 18-month visit and developmental diagnosis at 3–5 years: A prospective cohort study. *BMJ Paediatr Open, 6*(1). https://doi.org/https://doi.org/10.1136/bmjpo-2022-001524

Boyd, R. D., Welge, P., Sexton, D., & Miller, J. H. (1989). Concurrent validity of the Battelle Developmental Inventory: Relationship with the Bayley Scales in young children with known or suspected disabilities. *J Early Interv, 13*(1), 14-23. https://doi.org/10.1177/105381518901300103

Bradley-Johnson, S. (1997). Mullen Scales Of Early Learning. *34*(4), 379-382. https://doi.org/https://doi.org/10.1002/(SICI)1520-6807(199710)34:4<379::AID-PITS14>3.0.CO;2-E

Breaux, R. P., Griffith, S. F., & Harvey, E. A. (2016). Preschool neuropsychological measures as predictors of later attention deficit hyperactivity disorder. *J abnormal child psychol, 44*, 1455-1471. https://doi.org/https://doi.org/10.1007/s10802-016-0140-1

Brigance, A. H., & French, B., F. (2013). *Brigance Early Childhood Screens III*. North Billerica, MA: Curriculum Associates.

Brooks, B. L., Sherman, E. M., & Strauss, E. (2009). NEPSY-II: A developmental neuropsychological assessment. *Child Neuropsychol, 16*(1), 80-101. https://doi.org/https://doi.org/10.1080/09297040903146966

Brothers, K. B., Glascoe, F. P., & Robertshaw, N. S. (2008). PEDS: Developmental Milestones—an accurate brief tool for surveillance and screening. *Clin Pediatr, 47*(3), 271-279. https://doi.org/https://doi.org/10.1177/0009922807309419

Burdon, B. (1993). Australian Developmental Screening Test: Adst. *Australia: Harcourt Brace & Co Group (Australia) Pty and the Social-emotional Corporation*.

Burgess, D. B., Asher, K. N., Doucet, H. J., Reardon, K., & Daste, M.-L. R. (1984). Parent report as a means of administering the Prescreening Developmental Questionnaire: An evaluation study. *J dev behav pediatr, 5*(4), 195-200.

Burns, T. G., King, T. Z., & Spencer, K. S. (2013). Mullen Scales Of Early Learning: The utility in assessing children diagnosed with autism spectrum disorders, cerebral palsy, and epilepsy. *Appl Neuropsychol Child*, *2*(1), 33-42. https://doi.org/https://doi.org/10.1080/21622965.2012.682852

Cairney, J., Clinton, J., Veldhuizen, S., Rodriguez, C., Missiuna, C., Wade, T., Szatmari, P., & Kertoy, M. (2016). Evaluation of the revised Nipissing District Developmental Screening (NDDS) tool for use in general population samples of infants and children. *BMC pediatr*, *16*, 1-8. https://doi.org/https://doi.org/10.1186/s12887-016-0577-y

Caudle, S. E., Katzenstein, J. M., Oghalai, J. S., Lin, J., & Caudle, D. D. (2014). Nonverbal cognitive development in children with cochlear implants: Relationship between the Mullen Scales Of Early Learning and later performance on the Leiter International Performance Scales–Revised. *Assessment*, *21*(1), 119-128. https://doi.org/10.1177/1073191112437594

Cepanec, M., Lice, K., & Šimleša, S. (2012). Mother–father differences in screening for developmental delay in infants and toddlers. *J Commun Disord*, *45*(4), 255-262. https://doi.org/10.1016/j.jcomdis.2012.04.002

Chen, C.-Y., Xie, H., Clifford, J., Chen, C.-I., & Squires, J. (2018). Examining internal structures of a developmental measure using multidimensional item response theory. *J Early Interv*, *40*(4), 287-303. https://doi.org/10.1177/1053815118788063

Cheong, P.-L., Tsai, J.-M., Wu, Y.-T., Lu, L., Chiu, Y.-L., Shen, Y.-T., Li, Y.-J., Tsao, C.-H., Wang, Y.-C., & Chang, F.-M. (2022). Cultural adaptation and validation of Mullen Scales Of Early Learning in Taiwanese children with autism spectrum disorder, global developmental delay, and typically developing children. *Res dev disabil*, *122*, 104158. https://doi.org/https://doi.org/10.1016/j.ridd.2021.104158

Chong, K., Zhou, V., Tarazona, D., Tuesta, H., Velasquez-Hurtado, J., Sadeghi, R., & Llanos, F. (2017). ASQ-3 scores are sensitive to small differences in age in a Peruvian infant population. *Child Care Health Dev*, *43*(4), 556-565. https://doi.org/https://doi.org/10.1111/cch.12469

Cirelli, I., Bickle Graz, M., & Tolsa, J.-F. (2015). Comparison of Griffiths-II and Bayley-II tests for the developmental assessment of high-risk infants. *Infant Behav Dev*, *41*, 17-25. https://doi.org/10.1016/j.infbeh.2015.06.004

Clarkson, T., LeBlanc, J., DeGregorio, G., Vogel-Farley, V., Barnes, K., Kaufmann, W. E., & Nelson, C. A. (2017). Adapting the Mullen Scales Of Early Learning for a standardized measure of development in children with Rett syndrome. *Intellect Dev Disabil*, *55*(6), 419-431. https://doi.org/https://doi.org/10.1352/1934-9556-55.6.419

Colbert, A. M., Lamb, M. M., Asturias, E. J., Muñoz, F. M., Bauer, D., Arroyave, P., Hernández, S., Martínez, M. A., Paniagua-Avila, A., & Olson, D. (2020). Reliability and validity of an adapted and translated version of the Mullen Scales Of Early Learning (AT-MSEL) in rural Guatemala. *Child Care Health Dev, 46*(3), 327-335. https://doi.org/https://doi.org/10.1111/cch.12748

Coughlan, K. A. (2015). *Test review: Developmental Indicators for the Assessment of Learning, (dial-4), by c. Mardell-czudnowski & ds goldenberg*. Los Angeles, CA: SAGE Publications Sage CA.

Cronje, J. (2021). *Griffiths III technical report 2021*. H. Ltd.

Cronje, J. H., Green, E. M., & Stroud, L. A. (2022). Stability reliability of the Griffiths Scales Of Child Development (3rd edition). *Psychology, 13*, 353-360.

Cunha, A. C. B., Berkovits, M. D., & Albuquerque, K. A. (2018). Developmental assessment with young children: A systematic review of Battelle studies. *Infants & Young Children, 31*(1), 69-90. https://doi.org/10.1097/iyc.0000000000000106

Davis, J. L., & Matthews, R. N. (2010). NEPSY-II review: Korkman, m., kirk, u., & kemp, s.(2007). NEPSY—second edition (NEPSY-II). San Antonio, TX: Harcourt assessment. *J Psychoeduc Assess, 28*(2), 175-182. https://doi.org/https://doi.org/10.1177/0734282909346716

De-Andrés-Beltrán, B., Rodríguez-Fernández, Á. L., Güeita-Rodríguez, J., & Lambeck, J. (2015). Evaluation of the psychometric properties of the Spanish version of the Denver Developmental Screening Test II. *Eur J Pediatr, 174*(3), 325-329. https://doi.org/10.1007/s00431-014-2410-7

de Bildt, A., Kraijer, D., Sytema, S., & Minderaa, R. (2005). The psychometric properties of the Vineland Adaptive Behavior Scales in children and adolescents with mental retardation. *J autism dev disord, 35*, 53-62. https://doi.org/https://doi.org/10.1007/s10803-004-1033-7

Dempsey, A. G., Abrahamson, C. W., & Keller-Margulis, M. A. (2016). Developmental screening among children born preterm in a high-risk follow-up clinic. *J Pediatr Psychol, 41*(5), 573-581. https://doi.org/10.1093/jpepsy/jsv101

Doig, K. B., Macias, M. M., Saylor, C. F., Craver, J. R., & Ingram, P. E. (1999). The Child Development Inventory: A developmental outcome measure for follow-up of the high-risk infant. *J Pediatr, 135*(3), 358-362. https://doi.org/10.1016/S0022-3476(99)70134-4

Doove, B., Feron, J., Feron, F., van Os, J., & Drukker, M. (2019). Validation of short instruments assessing parental and caregivers' perceptions on child health and development for personalized prevention. *Clin Child Psychol Psychiatry, 24*(3), 608-630. https://doi.org/https://doi.org/10.1177/1359104518822673

Doskey, E. M., Lagunas, B., SooHoo, M., Lomax, A., & Bullick, S. (2013). Test review: C. Mardell & d. S. Goldenberg. Speed Developmental Indicators for the Assessment of Learning—fourth edition (speed dial-4). *J Psychoeduc Assess*, *31*(6), 611-616. https://doi.org/10.1177/0734282913484444

du Toit, M. N., Van der Linde, J., & Swanepoel, D. W. (2021). mHealth developmental screening for preschool children in low-income communities. *J Child Health Care, 25*(4), 573-586. https://doi.org/https://doi.org/10.1177/1367493520970012

Duyme, M., Zorman, M., Tervo, R., & Capron, C. (2011). French norms and validation of the Child Development Inventory (CDI): Inventaire du développement de l'enfant (ide). *Clin Pediatr*, *50*(7), 636-647. https://doi.org/10.1177/0009922811398390

Eadie, P. A., Ukoumunne, O., Skeat, J., Prior, M. R., Bavin, E., Bretherton, L., & Reilly, S. (2010). Assessing early communication behaviours: Structure and validity of the Communication and Symbolic Behaviour Scales—Developmental Profile (CSBS-DP) in 12-month-old infants. *Int J Lang Commun Disord, 45*(5), 572-585. https://doi.org/https://doi.org/10.3109/13682820903277944

Eck, G. P. (2011). *The Gesell Developmental Observation: Eight case studies in identifying special needs*. Dowling College.

Eernisse, E. R. (2017). Clinical Linguistic and Auditory Milestone Scale. *Encyclopedia of Autism Spectrum Disorders*. https://doi.org/https://doi.org/10.1007/978-1-4614-6435-8_1661-3

Eigsti, H. J., Chandler, L., Robinson, C., & Bodkin, A. W. (2010). A longitudinal study of outcome measures for children receiving early intervention services. *Pediatr Phys Ther*, *22*(3), 304-313. https://doi.org/https://doi.org/10.1097/PEP.0b013e3181e94464

El-Behadli, A. F., Neger, E. N., Perrin, E. C., & Sheldrick, R. C. (2015). Translations of developmental screening instruments: An evidence map of available research. *J dev behav pediatr*, *36*(6), 471-483. https://doi.org/10.1097/DBP.0000000000000193

Elbaum, B., Gattamorta, K. A., & Penfield, R. D. (2010). Evaluation of the Battelle Developmental Inventory, 2nd edition, screening test for use in states' child outcomes measurement systems under the individuals with disabilities education act. *J Early Interv*, *32*(4), 255-273. https://doi.org/10.1177/1053815110384723

Fäldt, A., Fabian, H., Dahlberg, A., Thunberg, G., Durbeej, N., & Lucas, S. (2021). Infant-Toddler Checklist identifies 18-month-old children with communication difficulties in the Swedish child healthcare setting. *Acta Paediatr*, *110*(5), 1505-1512. https://doi.org/https://doi.org/10.1111/apa.15696

Fantuzzo, J., Hightower, D., Grim, S., & Montes, G. (2002). Generalization of the Child Observation Record: A validity study for diverse samples of urban, low-income preschool children. *Early Childhood Research Quarterly*, *17*(1), 106-125. https://doi.org/https://doi.org/10.1016/S0885-2006(02)00131-X

Farmer, C., Adedipe, D., Bal, V., Chlebowski, C., & Thurm, A. (2020). Concordance of the Vineland Adaptive Behavior Scales, second and third editions. *J intellect disabil res*, *64*(1), 18-26. https://doi.org/https://doi.org/10.1111/jir.12691

Farmer, C., Golden, C., & Thurm, A. (2016). Concurrent validity of the Differential Ability Scales, second edition with the Mullen Scales of Early Learning in young children with and without neurodevelopmental disorders. *Child Neuropsychol*, *22*(5), 556-569. https://doi.org/10.1080/09297049.2015.1020775

Fauls, J. R., Thompson, B. L., & Johnston, L. M. (2020). Validity of the Ages and Stages Questionnaire to identify young children with gross motor difficulties who require physiotherapy assessment. *Dev Med Child Neurol*, *62*(7), 837-844. https://doi.org/https://doi.org/10.1111/dmcn.14480

Fleming, J. (1981). An evaluation of the use of the Denver Developmental Screening Test. *Nurs Res*, *30*(5), 290-293. https://doi.org/10.1097/00006199-198109000-00010

Foundation, H. E. R. (2013). Technical report of the COR Advantage validation study.

Frankenburg, W. K., Dodds, J., Archer, P., Shapiro, H., & Bresnick, B. (1992, Jan). The Denver II: A major revision and restandardization of the denver developmental screening test. *Pediatrics*, *89*(1), 91-97.

Frankenburg, W. K., Fandal, A. W., & Thornton, S. M. (1987). Revision of Denver Prescreening Developmental Questionnaire. *J Pediatr*, *110*(4), 653-657. https://doi.org/https://doi.org/10.1016/s0022-3476(87)80573-5

French, B., F. (2013). Brigance Screens III technical manual. https://oms.brigance.com/Reports/ScreensIII-Tech-Manual-04.pdf

Frisk, V., Montgomery, L., Boychyn, E., Young, R., McLachlan, D., & Neufeld, J. (2009). Why screening Canadian preschoolers for language delays is more difficult than it should be. *Infants Young Child*, *22*(4), 290-308. https://doi.org/https://doi.org/10.1097/IYC.0b013e3181bc4db6

Fulton, M. L., & D'Entremont, B. (2013). Utility of the Psychoeducational Profile-3 for assessing cognitive and language skills of children with autism spectrum disorders. *J autism dev disord*, *43*(10), 2460-2471. https://doi.org/10.1007/s10803-013-1794-y

Gao, X. (2008). *Young children's accountability data on language, literacy and pre-math areas: Validating authentic assessment* ProQuest Dissertations Publishing].

Garcia de Alba, R. (2007). *Using the Developmental Indicators for the Assessment of Learning-as a screener for young children: A comparison of the psychometric properties between the English and Spanish-speaking standardization samples* Texas A&M University].

Genteman, M. R. (1992). *A validity study of the AGS Early Screening Profiles with the Stanford-Binet fourth edition as criterion*

Girault, J. B., Langworthy, B. W., Goldman, B. D., Stephens, R. L., Cornea, E., Steven Reznick, J., Fine, J., & Gilmore, J. H. (2018). The predictive value of developmental assessments at 1 and 2 for intelligence quotients at 6. *Intelligence*, *68*, 58-65. https://doi.org/10.1016/j.intell.2018.03.003

Glascoe, F. P. (1998). *Collaborating with parents: Using Parents' Evaluation of Developmental Status in screening, surveillance, and promotion*. Ellsworth & Vandermeer Press.

Goh, S. K. Y., Tham, E. K. H., Magiati, I., Sim, L., Sanmugam, S., Qiu, A., Daniel, M. L., Broekman, B. F. P., & Rifkin-Graboi, A. (2017). Analysis of item-level bias in the Bayley-III language subscales: The validity and utility of standardized language assessment in a multilingual setting. *J Speech Lang Hear Res, 60*(9), 2663-2671. https://doi.org/10.1044/2017_JSLHR-L-16-0196

Goldberg, M. R., Dill, C. A., Shin, J. Y., & Nhan, N. V. (2009). Reliability and validity of the Vietnamese Vineland Adaptive Behavior Scales with preschool-age children. *Res dev disabil, 30*(3), 592-602. https://doi.org/https://doi.org/10.1016/j.ridd.2008.09.001

Grisham, J., Waddell, M., Crawford, R., & Toland, M. (2021). Psychometric properties of the Assessment, Evaluation, and Programming System for infants and children–third edition (aeps-3). *J Early Interv, 43*(1), 24-37. https://doi.org/10.1177/1053815120967359

Guddemi, M., Sambrook, A., Randel, B., & Selva, G. (2012). Gesell Developmental Observation-Revised and Gesell Early Screener technical report, ages 3-6. *New Haven, CT: Gesell Institute of Child Development*.

Gudmundsson, E. (2015, 2015/10/01/). The Toddler Language and Motor Questionnaire: A mother-report measure of language and motor development. *Res dev disabil, 45-46*, 21-31. https://doi.org/https://doi.org/10.1016/j.ridd.2015.07.007

Guedes, D. Z., Primi, R., & Kopelman, B. I. (2011). BINS validation – Bayley Neurodevelopmental Screener in Brazilian preterm children under risk conditions. *Infant Behav Dev*, *34*(1), 126-135. https://doi.org/10.1016/j.infbeh.2010.11.001

Guidubaldi, J., & Perry, J. D. (1984). Concurrent and predictive validity of the Battelle Development Inventory at the first grade level. *Educ psychol meas*, *44*(4), 977-985. https://doi.org/https://doi.org/10.1177/001316448444402

Haataja, L., McGready, R., Arunjerdja, R., Simpson, J. A., Mercuri, E., Nosten, F., & Dubowitz, L. (2002, Dec). A new approach for neurological evaluation of infants in resource-poor settings. *Ann Trop Paediatr*, *22*(4), 355-368. https://doi.org/10.1179/027249302125002029

Hamrick, L. R., Haney, A. M., Kelleher, B. L., & Lane, S. P. (2020). Using generalizability theory to evaluate the comparative reliability of developmental measures in neurogenetic syndrome and low-risk populations. *J Neurodev Disord*, *12*(1), 1-15. https://doi.org/https://doi.org/10.1186/s11689-020-09318-1

Hamrick, L. R., & Tonnsen, B. L. (2019). Validating and applying the CSBS-ITC in neurogenetic syndromes. *Am J Intellect Dev Disabil*, *124*(3), 263-285. https://doi.org/https://doi.org/10.1352/1944-7558-124.3.263

Hanlon, C., Medhin, G., Worku, B., Tomlinson, M., Alem, A., Dewey, M., & Prince, M. (2016). Adapting the Bayley Scales of Infant and Toddler Development in Ethiopia: Evaluation of reliability and validity. *Child Care Health Dev*, *42*(5), 699-708. https://doi.org/10.1111/cch.12371

Harrison, P. L., Kaufman, A. S., Kaufman, N. L., Bruinicks, R. H., Rynders, J., Ilmer, S., Sparrow, S. S., & Cicchetti, D. V. (1990). *AGS Early Screening Profiles*. American Guidance Service.

Hess, C. R., Papas, M. A., & Black, M. M. (2004). Use of the Bayley Infant Neurodevelopmental Screener with an environmental risk group. *J Pediatr Psychol*, *29*(5), 321-330. https://doi.org/10.1093/jpepsy/jsh036

Hilton-Mounger, A. (2011). Battelle Developmental Inventory: 2nd edition. In S. Goldstein & J. A. Naglieri (Eds.), *Encyclopedia of child behavior and development*. Boston, MA: Springer US. https://doi.org/10.1007/978-0-387-79061-9_290

Hsia, T.-h. (1993). *Evaluating the psychometric properties of the Assessment, Evaluation, and Programming System for 3 to 6 years: AEPS test 3 to 6 years (AEPS test)* ProQuest Dissertations Publishing].

Huang, C.-Y., Tung, L.-C., Chou, Y.-T., Chou, W., Chen, K.-L., & Hsieh, C.-L. (2018). Improving the utility of the fine motor skills subscale of the Comprehensive Developmental Inventory for Infants and Toddlers: A computerized adaptive test. *Disabil rehabil, 40*(23), 2803-2809. https://doi.org/10.1080/09638288.2017.1356385

Huang, C.-Y., Tung, L.-C., Chou, Y.-T., Wu, H.-M., Chen, K.-L., & Hsieh, C.-L. (2018). Development of a computerized adaptive test of children's gross motor skills. *Arch phys med rehabil*, *99*(3), 512-520. https://doi.org/https://doi.org/10.1016/j.apmr.2017.07.017

Huisenga, D., La Bastide-Van Gemert, S., Van Bergen, A., Sweeney, J., & Hadders-Algra, M. (2021). Developmental outcomes after early surgery for complex congenital heart disease: A systematic review and meta-analysis. *Dev Med Child Neurol*, *63*(1), 1-19. https://doi.org/10.1111/dmcn.14512

Hwang, A. W., Weng, L. J., & Liao, H. f. (2010). Construct validity of the Comprehensive Developmental Inventory for Infants and Toddlers. *Pediatr int*, *52*(4), 598-606. https://doi.org/https://doi.org/10.1111/j.1442-200X.2010.03102.x

Hwang, C. P. (1987, 1987). Caesarean childbirth in Sweden: Effects on the mother and father-infant relationship. *Infant Ment Health J*, *8*(2), 91-99. https://doi.org/https://doi.org/10.1002/1097-0355(198722)8:2<91::AID-IMHJ2280080202>3.0.CO;2-D

Ireton, H. (1990). Child Development Review—parent questionnaire. *Minneapolis, MN: Behavior Science Systems*.

Ireton, H. (1992). *Child Development Inventory manual*. Behavior Science Systems.

Ireton, H. (1994). *How to use the "Infant Development Inventory"*. Behavior Science Systems. Retrieved February 6 from https://static1.squarespace.com/static/562e8e0ae4b09db47d931eb9/t/598a2eb3f5e23155afc054a5/1502228154319/IDI+Instructions-3.pdf

Ireton, H. (1996). The Child Development Review: Monitoring children's development using parents' and pediatricians' observations. *Infants Young Child*, *9*(1), 42-52. https://doi.org/10.1097/00001163-199607000-00006

James, S., Ziviani, J., & Boyd, R. (2014). A systematic review of activities of daily living measures for children and adolescents with cerebral palsy. *Dev Med Child Neurol, 56*(3), 233-244. https://doi.org/https://doi.org/10.1111/dmcn.12226

Johnson, J., & Macy, M. (2019). An introduction to the AEPS-3 and results of a field test study. *Education and New Developments*, 59-63. https://doi.org/10.36315/2019v1end013

Johnson, M. O. (1997). *Mother-child interaction in the presence of maternal human immunodeficiency virus infection* (Publication Number 9726763) [Ph.D., University of South Carolina with Medical University of South Carolina]. ProQuest Dissertations & Theses Global. United States -- South Carolina.

Karam, F., Sheehy, O., Huneau, M.-C., Chambers, C., Fraser, W. D., Johnson, D., Kao, K., Martin, B. Z., Riordan, S. H., & Roth, M. (2015). The ASQ and R-PDQ telephone-administered validation within the otis antidepressant in pregnancy study. *Psychol Assess, 27*(4), 1507. https://doi.org/https://doi.org/10.1037/pas0000084

Katz, E. R. (2016). *The predictive validity of a kindergarten screener above demographic, ecological, and school variables* St. John's University]. ProQuest.

Korkman, M., Kirk, U., & Kemp, S. (2007). *NEPSY-second ediution (NEPSY-II).* San Antonio, TX: Harcourt Assessment.

Koura, G. K., Boivin, M. J., Davidson, L. L., Ouédraogo, S., Zoumenou, R., Alao, M. J., Garcia, A., Massougbodji, A., Cot, M., & Bodeau-Livinec, F. (2013). Usefulness of child development assessments for low-resource settings in francophone Africa. *J Dev Behav Pediatr, 34*(7). https://doi.org/https://doi.org/10.1097/DBP.0b013e31829d211c

Krivitzky, L., Bosenbark, D. D., Ichord, R., Jastrzab, L., & Billinghurst, L. (2019). Brief report: Relationship between performance testing and parent report of attention and executive functioning profiles in children following perinatal arterial ischemic stroke. *Child Neuropsychol, 25*(8), 1116-1124. https://doi.org/https://doi.org/10.1080/09297049.2019.1588957

Krogh, M. T., & Væver, M. S. (2019). A longitudinal study of the predictive validity of the Bayley-III scales and subtests. *Eur J Dev Psychol, 16*(6), 727-738. https://doi.org/10.1080/17405629.2018.1485563

Kumar, R., Shankar, K., Kush, V., Kumar, C., Bhave, A., & Agarwal, V. (2016). Adaptation: Vineland Adaptive Behavior Scale for 3–9 year-old Indian children. *Int J Disabil Hum Dev, 15*(1), 49-55. https://doi.org/https://doi.org/10.1515/ijdhd-2014-0026

L'Hotta, A. J., Hoyt, C. R., Lindsey, T., Abel, R. A., Chang, C. H., & King, A. A. (2020). Validation of the fine motor subtest of the Bayley-III with children with sickle cell disease using rasch analysis. *Child Care Health Dev*, *46*(5), 576-584. https://doi.org/10.1111/cch.12795

LaForte, E. M. (2014). *Validation of score interpretations for the BDI-2 using rasch methodology*. ProQuest Dissertations Publishing]. Loyola University.

Larg, R., Graf, S., Kundu, S., Hunziker, U., & Molinari, L. (1990). Predicting developmental outcome at school age from infant tests of normal, at-risk and retarded infants. *Dev Med Child Neurol*, *32*(1), 30-45. https://doi.org/https://doi.org/10.1111/j.1469-8749.1990.tb08464.x

Larson, A. L. (2016). Language screening for infants and toddlers: A literature review of four commercially available tools. *Commun Disord Q*, *38*(1), 3-12. https://doi.org/10.1177/1525740115627420

Lasee, M. J., & Smith, D. K. (1991). Relationships between the K-ABC and the Early Screening Profiles.

Leonard, C. H., Piecuch, R. E., & Cooper, B. A. (2001, Jan-Feb). Use of the Bayley Infant Neurodevelopmental Screener with low birth weight infants. *J Pediatr Psychol*, *26*(1), 33-40. https://doi.org/10.1093/jpepsy/26.1.33

Leung, C., Cheung, J., Lau, V., & Lam, C. (2011, 2011/11/01/). Development of the Preschool Developmental Assessment Scale (PDAS) on children's social development. *Res Dev Disabil*, *32*(6), 2511-2518. https://doi.org/https://doi.org/10.1016/j.ridd.2011.07.009

Leung, C., Mak, R., Lau, V., Cheung, J., & Lam, C. (2013). The validation of a scale to measure cognitive development in Chinese preschool children. *Res Dev Disabil*, *34*(7), 2257-2267. https://doi.org/10.1016/j.ridd.2013.04.003

Li, Z. (2016). *Longitudinal analysis for ordinal data through multilevel and item response modeling: Applications to child observation record (cor)* ProQuest Dissertations Publishing].

Liao, H.-F., & Pan, Y.-L. (2005). Test–retest and inter-rater reliability for the Comprehensive Developmental Inventory for Infants and Toddlers diagnostic and screening tests. *Early Hum Dev, 81*(11), 927-937. https://doi.org/https://doi.org/10.1016/j.earlhumdev.2005.07.008

Liao, H.-F., Yao, G., & Wang, T.-M. (2008). Concurrent validity in Taiwan of the Comprehensive Developmental Inventory for Infants and Toddlers who were full-term infants. *Percept Motor Skills, 107*(1), 29-44. https://doi.org/https://doi.org/10.2466/pms.107.1.29-44

Lin, C.-S., & Chiu, C.-H. (2014). Adaptation of the chinese edition of the CSBS-DP: A cross-cultural comparison of prelinguistic development between Taiwanese and American toddlers. *Res Dev Disabil, 35*(5), 1042-1050. https://doi.org/https://doi.org/10.1016/j.ridd.2014.01.034

Lin, L.-Y., Tu, Y.-F., Yu, W.-H., Ho, M.-H., & Wu, P.-M. (2020). Investigation of fine motor performance in children younger than 36-month-old using PDMS-2 and Bayley-III. *Eur J Dev Psychol, 17*(5), 746-760. https://doi.org/10.1080/17405629.2020.1732917

Lopes, S., Graça, P., Teixeira, S., Serrano, A. M., & Squires, J. (2015). Psychometric properties and validation of Portuguese version of Ages & Stages Questionnaires: 9, 18 and 30 questionnaires. *Early Hum Dev*, *91*(9), 527-533. https://doi.org/https://doi.org/10.1016/j.earlhumdev.2015.06.006

Lopez Boo, F., Cubides Mateus, M., & Llonch Sabatés, A. (2020). Initial psychometric properties of the Denver II in a sample from northeast Brazil. *Infant Behav Dev, 58*, 101391. https://doi.org/10.1016/j.infbeh.2019.101391

Luiz, D. M., Foxcroft, C. D., & Stewart, R. (2001). The construct validity of the Griffiths scales of mental development. *Child Care Health Dev, 27*(1), 73-83. https://doi.org/10.1046/j.1365-2214.2001.00158.x

Luiz, D. M., Foxcroft, C. D., & Tukulu, A. N. (2004). The Denver II scales and the Griffiths scales of mental development: A correlational study. *J Child Adolesc Ment Health, 16*(2), 77-81. https://doi.org/https://doi.org/10.2989/17280580409486573

Luttikhuizen dos Santos, E. S., de Kieviet, J. F., Königs, M., van Elburg, R. M., & Oosterlaan, J. (2013). Predictive value of the Bayley Scales of Infant Development on development of very preterm/very low birth weight children: A meta-analysis. *Early Hum Dev, 89*(7), 487-496. https://doi.org/10.1016/j.earlhumdev.2013.03.008

Ma, P. S. (2012). *Children with autism in Taiwan and the United States: Parental stress, parent-child relationships, and the reliability of a child development inventory* ProQuest Dissertations Publishing]. University of North Texas.

Maleka, B. K., Van Der Linde, J., Glascoe, F. P., & Swanepoel, D. W. (2016). Developmental screening—evaluation of an m-health version of the Parents Evaluation Developmental Status tools. *Telemed J E Health, 22*(12), 1013-1018. https://doi.org/https://doi.org/10.1089/tmj.2016.0007

Månsson, J., Stjernqvist, K., Serenius, F., Ådén, U., & Källén, K. (2019). Agreement between Bayley-III Measurements and WISC-IV measurements in typically developing children. *J Psychoeduc Assess, 37*(5), 603-616. https://doi.org/10.1177/0734282918781431

Mardell-Czudnowski, C., Chien-Hou, H., & Tien-Miau, W. (1986). Cross-cultural adaptation of a developmental test (DIAL-R) for young children in Taiwan. *J Cross Cult Psychol*, *17*(4), 475-492. https://doi.org/https://doi.org/10.1177/0022002186017004006

Mardell, C., & Goldenberg, D. S. (1976). The predictive validation of a pre-kindergarten screening test. https://eric.ed.gov/?id=ED135157

Mardell, C., & Goldenberg, D. S. (2011). *Developmental Indicators for the Assessment of Learning, fourth edition (DIAL-4) manual*. Bloomington, MN: Pearson.

Marks, K. P., Madsen Sjö, N., & Wilson, P. (2019). Comparative use of the Ages and Stages Questionnaires in the USA and Scandinavia: A systematic review. *Dev Med Child Neurol, 61*(4), 419-430. https://doi.org/https://doi.org/10.1111/dmcn.14044

Martínez-Nadal, S., Schonhaut, L., Armijo, I., & Demestre, X. (2021). Predictive value of the Ages and Stages Questionnaire® for school performance and school intervention in late preterm- and term-born children. *Child Care Health Dev, 47*(1), 103-111. https://doi.org/10.1111/cch.12814

Mattar, J. W., & Arouri, Y. M. (2017). A pilot study for extracting psychometric properties of an adapted Jordanian version of the Parents' Evaluation of Developmental Milestones (PEDS: DM). *Int J of Spec Educ*, *32*(3), 618-629.

McCarthy, A. M., Wehby, G. L., Barron, S., Aylward, G. P., Castilla, E. E., Javois, L. C., Goco, N., & Murray, J. C. (2012). Application of neurodevelopmental screening to a sample of South American infants: The Bayley Infant Neurodevelopmental Screener (BINS). *Infant Behav Dev*, *35*(2), 280-294. https://doi.org/10.1016/j.infbeh.2011.12.003

McDonald, J., & Milne, S. (2013). Australian Developmental Screening Test. *J Paediatr Child Health*, *49*(3), 255-255. https://doi.org/10.1111/jpc.12125

McHenry, M. S., Oyungu, E., Yang, Z., Hines, A. C., Ombitsa, A. R., Vreeman, R. C., Abubakar, A., & Monahan, P. O. (2021). Cultural adaptation of the Bayley Scales of Infant and Toddler Development, 3rd edition for use in Kenyan children aged 18–36 months: A psychometric study. *Res Dev Disabil*, *110*, 103837. https://doi.org/10.1016/j.ridd.2020.103837

McIntosh, D. E., Gibney, L., Quinn, K., & Kundert, D. (2000). Concurrent validity of the Early Screening Profiles and the Differential Ability Scales with an at-risk preschool sample. *Psychol Sch*, *37*(3), 201-207. https://doi.org/https://doi.org/10.1002/(SICI)1520-6807(200005)37:3<201::AID-PITS1>3.0.CO;2-A

McLean, M., McCormick, K., Baird, S., & Mayfield, P. (1987). Concurrent validity of the Battelle Developmental Inventory screening test. *Diagnostique*, *13*(1), 10-20. https://doi.org/https://doi.org/10.1177/073724778701300102

Meisels, S., Marsden, D., Wiske, M., & Henderson, L. (2008). Early Screening Inventory-revised, 2008 edition (esi-r). *AnnArbor, MI: Rebus*.

Meisels, S. J., Henderson, L. W., Liaw, F.-r., Browning, K., & Ten Have, T. (1993). New evidence for the effectiveness of the Early Screening Inventory. *Early Child Res Q, 8*(3), 327-346. https://doi.org/https://doi.org/10.1016/S0885-2006(05)80071-7

Mendonça, B., Sargent, B., & Fetters, L. (2016). Cross-cultural validity of standardized motor development screening and assessment tools: A systematic review. *Dev Med Child Neurol, 58*(12), 1213-1222. https://doi.org/10.1111/dmcn.13263

Mirrett, P. L., Bailey, D. B., Roberts, J. E., & Hatton, D. D. (2004). Developmental screening and detection of developmental delays in infants and toddlers with fragile x syndrome. *J Dev Behav Pediatr, 25*(1), 21-27. https://doi.org/10.1097/00004703-200402000-00004

Montgomery, M. L., Saylor, C. F., Bell, N. L., Macias, M. M., Charles, J. M., & Pappu Katikaneni, L. D. (1999). Use of the Child Development Inventory to screen high-risk populations. *Clin Pediatr, 38*(9), 535-539. https://doi.org/10.1177/000992289903800906

Morris, J., Perkins, D., Sarkozy, V., Moline, A., Zwi, K., & Williams, K. (2012). Performance of the Australian Developmental Screening Test in a clinical setting: Australian Developmental Screening Test. *J Paediatr Child Health, 48*(11), 1004-1009. https://doi.org/10.1111/j.1440-1754.2012.02588.x

Moyal, N. (2010). *The Battelle Developmental Inventory, 2nd edition: A study of concurrent validity and stability in young children with known disabilities* ProQuest Dissertations Publishing]. Fairleigh Dickinson University.

Mullen, E. M. (1995). *Mullen Scales Of Early Learning*. Circle Pines, MN: AGS

Musser, C. E. (2001). *A study of the consistency of congruence between parent and professional estimates of a preschool child's developmental status*. ProQuest Dissertations Publishing].

Muthusamy, S., Wagh, D., Tan, J., Bulsara, M., & Rao, S. (2022). Utility of the Ages and Stages Questionnaire to identify developmental delay in children aged 12 to 60 months: A systematic review and meta-analysis. *JAMA Pediatr, 176*(10), 980-989. https://doi.org/10.1001/jamapediatrics.2022.3079

Newborg, J. (2005). *Battelle Developmental Inventory, 2nd edition examiners manual*. Itasca, IL: Riverside.

Nguyen, K. V. H. (2017). *Adaptation of the Bayley Scales of Infant and Toddler Development, third edition (Bayley-III) for Vietnam: A preliminary study*. ProQuest Dissertations Publishing]. St. John's University.

Nipissing District Developmental Screen Intellectual Property Association. (2000). Nipissing District Developmental Screen. *North Bay, Ontario, Canada: Nipissing District Developmental Screen*.

Noh, J. (2005). *Examining the psychometric properties of the second edition of the Assessment, Evaluation, and Programming System for three to six years: AEPS test 2nd edition (3–6)*. ProQuest Dissertations Publishing].

Nordahl-Hansen, A., Kaale, A., & Ulvund, S. E. (2014). Language assessment in children with autism spectrum disorder: Concurrent validity between report-based assessments and direct tests. *Res Autism Spectr Disord*, *8*(9), 1100-1106. https://doi.org/https://doi.org/10.1016/j.rasd.2014.05.017

Novak, I., Morgan, C., Adde, L., Blackman, J., Boyd, R. N., Brunstrom-Hernandez, J., Cioni, G., Damiano, D., Darrah, J., Eliasson, A.-C., de Vries, L. S., Einspieler, C., Fahey, M., Fehlings, D., Ferriero, D. M., Fetters, L., Fiori, S., Forssberg, H., Gordon, A. M., Greaves, S., Guzzetta, A., Hadders-Algra, M., Harbourne, R., Kakooza-Mwesige, A., Karlsson, P., Krumlinde-Sundholm, L., Latal, B., Loughran-Fowlds, A., Maitre, N., McIntyre, S., Noritz, G., Pennington, L., Romeo, D. M., Shepherd, R., Spittle, A. J., Thornton, M., Valentine, J., Walker, K., White, R., & Badawi, N. (2017). Early, accurate diagnosis and early intervention in cerebral palsy: Advances in diagnosis and treatment. *JAMA Pediatr*, *171*(9), 897-907. https://doi.org/10.1001/jamapediatrics.2017.1689

O'Meagher, S., Norris, K., Kemp, N., & Anderson, P. (2019). Examining the relationship between performance-based and questionnaire assessments of executive function in young preterm children: Implications for clinical practice. *Child Neuropsychol*, *25*(7), 899-913. https://doi.org/https://doi.org/10.1080/09297049.2018.1531981

Obrzut, J. E., Bolocofsky, D. N., Heath, C. P., & Jones, M. J. (1981). An investigation of the DIAL as a pre-kindergarten screening instrument. *Educ Psychol Meas*, *41*(4), 1231-1241. https://doi.org/10.1177/001316448104100431

Olvera Astivia, O. L., Forer, B., Dueker, G. L., Cowling, C., & Guhn, M. (2017). The Ages And Stages Questionnaire: Latent factor structure and growth of latent mean scores over time. *Early Hum Dev*, *115*, 99-109. https://doi.org/10.1016/j.earlhumdev.2017.10.002

Ortiz-León, S., Granados-Rojas, A., Cavazos-Olivo, J., Benito-Avendaño, L. D., Obregón-García, V. H., Duran-Avendaño, X. M., Ramírez-Pérez, R. V., Cárdenas-Medina, J. A., Vargas-Terréz, B. E., & Luna-Guevara, G. (2018). Internal and inter-rater reliability of the ASQ-3 in Mexican preschoolers. *Salud Ment*, *41*(2), 65-72. https://doi.org/10.17711/SM.0185-3325.2018.011

Parikh, C., Iosif, A.-M., & Ozonoff, S. (2021). Brief report: Use of the Infant–Toddler Checklist in infant siblings of children with autism spectrum disorder. *J Autism Dev Disord*, *51*(3), 1007-1012. https://doi.org/10.1007/s10803-020-04468-6

Pederson, D. R., Evans, B., Chance, G. W., Bento, S., & Fox, A. M. (1988). Predictors of one-year developmental status in low birth weight infants. *J Dev Behav Pediatr*, *9*(5), 287-292.

Pendergast, L. L., Schaefer, B. A., Murray-Kolb, L. E., Svensen, E., Shrestha, R., Rasheed, M. A., Scharf, R. J., Kosek, M., Vasquez, A. O., Maphula, A., Costa, H., Rasmussen, Z. A., Yousafzai, A., Tofail, F., & Seidman, J. C. (2018). Assessing development across cultures: Invariance of the Bayley-III scales across seven international mal-ed sites. *Sch Psychol Q*, *33*(4), 604-614. https://doi.org/10.1037/spq0000264

Rajendran, K., O'Neill, S., Marks, D. J., & Halperin, J. M. (2015). Latent profile analysis of neuropsychological measures to determine preschoolers' risk for ADHD. *J Child Psychol Psychiatry, 56*(9), 958-965. https://doi.org/https://doi.org/10.1111/jcpp.12434

Ranjitkar, S., Kvestad, I., Strand, T. A., Ulak, M., Shrestha, M., Chandyo, R. K., Shrestha, L., & Hysing, M. (2018). Acceptability and reliability of the Bayley Scales of Infant and Toddler Development-III among children in Bhaktapur, Nepal. *Frontiers in Psychology, 9*, 1-10. https://doi.org/10.3389/fpsyg.2018.01265

Reyes, A., Pacifico, R., Benitez, B., Villanueva-Uy, E., Lam, H., & Ostrea Jr, E. M. (2010). Use of the Griffiths Mental Development Scales in an agro-industrial province in the Philippines. *Child Care Health Dev, 36*(3), 354-360. https://doi.org/https://doi.org/10.1111/j.1365-2214.2010.01080.x

Rodriguez, V. J., Zegarac, M., La Barrie, D. L., Parrish, M. S., Matseke, G., Peltzer, K., & Jones, D. L. (2020). Validation of the Bayley Infant Neurodevelopmental Screener among HIV-exposed infants in rural South Africa. *J Acquir Immune Defic Syndr, 85*(4), 507-516. https://doi.org/10.1097/QAI.0000000000002479

Rosenbaum, M. (1981). Assessing children's development in a lower socioeconomic population.

Rosenbaum, M. S., Chua-Lim, C., Wilhite, J., & Mankad, V. N. (1983). Applicability of the Denver Prescreening Developmental Questionnaire in a low-income population. *Pediatrics, 71*(3), 359-363.

Rossman, M. J., Hyman, S. L., Rorabaugh, M. L., Berlin, L. E., Allen, M. C., & Modlin, J. F. (1994). The CAT/CLAMS assessment for early intervention services. *Clin Pediatr*, *33*(7), 404-409. https://doi.org/https://doi.org/10.1177/000992289403300705

Rousseau, M., Dionne, C., Savard, R. T., Schonhaut, L., & Londono, M. (2021). Translation and cultural adaptation of the Ages and Stages Questionnaires (ASQ) worldwide: A scoping review. *J Dev Behav Pediatr*, *42*(6), 490-501. https://doi.org/10.1097/DBP.0000000000000940

Rubio-Codina, M., Araujo, M. C., Attanasio, O., Muñoz, P., & Grantham-McGregor, S. (2016). Concurrent validity and feasibility of short tests currently used to measure early childhood development in large scale studies. *PLoS One*, *11*(8), e0160962. https://doi.org/10.1371/journal.pone.0160962

Rubio-Codina, M., Grantham-McGregor, S., & Tran, T. D. (2020). Predictive validity in middle childhood of short tests of early childhood development used in large scale studies compared to the Bayley-iii, the family care indicators, height-for-age, and stunting: A longitudinal study in Bogota, Colombia. *PLoS One*, *15*(4), e0231317. https://doi.org/10.1371/journal.pone.0231317

Saleh, R. M. A., & Smadi, J. M. (2017). The efficacy of arabic version of the Developmental Assessment of Young Children second edition (DAYC-2) scale in detecting developmental delay among Jordanian children aged birth to 71 months. *Int Educ Stud*, *10*(4), 113-132. https://doi.org/10.5539/IES.V10N4P113

Scattone, D., Raggio, D. J., & May, W. (2011). Comparison of the Vineland Adaptive Behavior Scales, and the Bayley Scales of Infant and Toddler Development. *Psychol Rep*, *109*(2), 626-634. https://doi.org/https://doi.org/10.2466/03.10.PR0.109.5.626-634

Schatz, J., McClellan, C. B., Puffer, E. S., Johnson, K., & Roberts, C. W. (2008). Neurodevelopmental screening in toddlers and early preschoolers with sickle cell disease. *J Child Neurol*, *23*(1), 44-50. https://doi.org/10.1177/0883073807307982

Schonhaut, L., Pérez, M., Armijo, I., & Maturana, A. (2020). Comparison between Ages & Stages Questionnaire and Bayley scales, to predict cognitive delay in school age. *Early Hum Dev*, *141*, 104933. https://doi.org/10.1016/j.earlhumdev.2019.104933

Sekino, Y., & Fantuzzo, J. (2005). Validity of the Child Observation Record: An investigation of the relationship between COR dimensions and social-emotional and cognitive outcomes for Head Start children. *J Psychoeduc Assess*, *23*(3), 242-260. https://doi.org/https://doi.org/10.1177/073428290502300304

Shahshahani, S., Sajedi, F., Azari, N., Vameghi, R., Kazemnejad, A., & Tonekaboni, S.-H. (2011). Evaluating the validity and reliability of PDQ-II and comparison with DDST-II for two step developmental screening. *Iran J Pediatr*, *21*(3), 343-349.

Shahshahani, S., Vameghi, R., Azari, N., Sajedi, F., & Kazemnejad, A. (2010). Validity and reliability determination of Denver Developmental Screening Test-II in 0-6 year–olds in Tehran. *Iran J Pediatr*, *20*(3), 313.

Sheldrick, R. C., Marakovitz, S., Garfinkel, D., Carter, A. S., & Perrin, E. C. (2020). Comparative accuracy of developmental screening questionnaires. *JAMA Pediatr*, *174*(4), 366-374. https://doi.org/https://doi.org/10.1001/jamapediatrics.2019.6000

Shrestha, M., Strand, T. A., Ulak, M., Chandyo, R. K., Ranjitkar, S., Hysing, M., Shrestha, L., & Kvestad, I. (2019). The feasibility of the Ages and Stages Questionnaire for the assessment of child development in a community setting in Nepal. *Child Care Health Dev*, *45*(3), 394-402. https://doi.org/https://doi.org/10.1111/cch.12654

Siegle, C. B. H., & dos Santos Cardoso de Sá, C. (2018). Concurrent validity between instruments of assessment of motor development in infants exposed to HIV. *Infant Behav Dev*, *50*, 198-206. https://doi.org/10.1016/j.infbeh.2018.01.005

Simpson, S., Eadie, T., Khoo, S. T., Titmuss, A., Maple-Brown, L. J., Thompson, R., Wunungmurra, A., Jeyaseelan, D., Dunham, M., & D'Aprano, A. (2021). The ASQ-TRAK: Validating a culturally adapted developmental screening tool for Australian Aboriginal children. *Early Hum Dev*, *163*, 105481. https://doi.org/https://doi.org/10.1016/j.earlhumdev.2021.105481

Singh, A., Yeh, C. J., & Boone Blanchard, S. (2017). Ages and stages Questionnaire: A global screening scale. *Bol Med Hosp Infant*, *74*(1), 5-12. https://doi.org/10.1016/j.bmhime.2016.07.001

Sipes, M., Matson, J. L., & Turygin, N. (2011). The use of the Battelle Developmental Inventory-second edition (BDI-2) as an early screener for autism spectrum disorders. *Dev Neurorehabil*, *14*(5), 310-314. https://doi.org/10.3109/17518423.2011.598477

Small, J. W., Hix-Small, H., Vargas-Baron, E., & Marks, K. P. (2019). Comparative use of the Ages and Stages Questionnaires in low- and middle-income countries. *Dev Med Child Neurol*, *61*(4), 431-443. https://doi.org/10.1111/dmcn.13938

Smith, D. K. (1990). Test-retest reliability of the AGS Early Screening Profiles.

Smith, D. K. (1991). The Early Screening Profiles: A stability study.

Snow, J. H. (1995). Book review: AGS Early Screening Profiles. *J Psychoeduc Assess*, *13*(1), 101-104. https://doi.org/https://doi.org/10.1177/073428299501300108

Snyder, P., Lawson, S., Thompson, B., Stricklin, S., & Sexton, D. (1993). Evaluating the psychometric integrity of instruments used in early intervention research: The Battelle Developmental Inventory. *Top Early Child Spec Educ*, *13*(2), 216-232. https://doi.org/https://doi.org/10.1177/027112149301300209

Soysal, A. S., Gucuyener, K., Ergenekon, E., Turan, Ö., Koc, E., Turkyılmaz, C., Önal, E., & Atalay, Y. (2014). The prediction of later neurodevelopmental status of preterm infants at ages 7 to 10 years using the Bayley Infant Neurodevelopmental Screener. *J Child Neurol*, *29*(10), 1349-1355. https://doi.org/10.1177/0883073813520495

Spagnola, K. T. Q. (2009). *Assessing the stability of the predictive validity of age of entry and the Developmental Indicators for the Assessment of Learning-(DIAL-3) on school achievement* St. John's University].

Sparrow, S. S., Cicchetti, D. V., & Saulnier, C. A. (2016). *Vineland-3: Vineland Adaptive Behavior Scales*. PsychCorp.

Spittle, A. J., Doyle, L. W., & Boyd, R. N. (2008). A systematic review of the clinimetric properties of neuromotor assessments for preterm infants during the first year of life. *Dev Med Child Neurol*, *50*(4), 254-266. https://doi.org/10.1111/j.1469-8749.2008.02025.x

Squires, J., Bricker, D. D., & Twombly, E. (2009). *Ages & Stages Questionnaires*. Baltimore, MD: Paul H. Brookes.

Squires, J., Twombly, E., Bricker, D., & Potter, L. (2009). *Ages & Stages Questionnaires, third edition (ASQ-3) user's guide*. Paul H. Brookes Publishing Company.

Stroud, L., Foxcroft, C., Green, E., Bloomfield, S., Conje, J., Hurter, K., Lane, H., Marais, R., Marx, C., McAlinden, P., O'Connell, R., Paradice, R., & Venter, D. (2016). *Griffiths scales of child development (3rd edition) part 1: Overview, development, and psychometric properties*. Oxford, United Kingdom: Hogrefe Limited.

Sutcliffe, A. G., Soo, A., & Barnes, J. (2010). Predictive value of developmental testing in the second year for cognitive development at five years of age. *Pediatr Rep*, *2*(2), e15. https://doi.org/https://doi.org/10.4081/pr.2010.e15

Swartzmiller, M. D. (2014). Test review: Developmental Assessment Of Young Children–second edition (DAYC-2). *Journal of Psychoeducational Assessment*, *32*(6), 577-580. https://doi.org/10.1177/0734282913518380

Swineford, L. B., Guthrie, W., & Thurm, A. (2015). Convergent and divergent validity of the Mullen Scales of Early Learning in young children with and without autism spectrum disorder. *Psychol Assess*, *27*(4), 1364. https://doi.org/https://doi.org/10.1037/pas0000116

Toh, T. H., Lim, B. C., Bujang, M. A. B., Haniff, J., Wong, S. C., & Abdullah, M. R. (2017). Mandarin Parents' Evaluation of Developmental Status in the detection of delays. *Pediatr Int*, *59*(8), 861-868. https://doi.org/10.1111/ped.13325

Torras Mañá, M., Gómez-Morales, A., González-Gimeno, I., Fornieles Deu, A., & Brun-Gasca, C. (2016). Assessment of cognition and language in the early diagnosis of autism spectrum disorder : Usefulness of the Bayley Scales of Infant and Toddler Development, third edition. *J Intellect Disabil Res*, *60*(5), 502-511. https://doi.org/https://doi.org/10.1111/jir.12291

Tsai, Y.-P., Tung, L.-C., Lee, Y.-C., Wang, Y.-L., Yen, Y.-S., & Chen, K.-L. (2016). Selecting score types for longitudinal evaluations: The responsiveness of the Comprehensive Developmental Inventory for Infants and Toddlers in children with developmental disabilities. *Neuropsychiatr Dis Treat*, 1103-1109. https://doi.org/https://doi.org/10.2147/NDT.S99171

Tso, W. W. Y., Wong, V. C. N., Xia, X., Faragher, B., Li, M., Xu, X., Ao, L., Zhang, X., Jiao, F. Y., Du, K., Shang, X., Wong, P. T. Y., & Challis, D. (2018). The Griffiths Development Scales-Chinese (GDS-C): A cross-cultural comparison of developmental trajectories between Chinese and British children. *Child Care Health Dev*, *44*(3), 378-383. https://doi.org/10.1111/cch.12548

Tuerk, C., Anderson, V., Bernier, A., & Beauchamp, M. H. (2021). Social competence in early childhood: An empirical validation of the social model. *J Neuropsychol*, *15*(3), 477-499. https://doi.org/https://doi.org/10.1111/jnp.12230

van den Heuvel, M., Borkhoff, C. M., Koroshegyi, C., Zabih, W., Reijneveld, S. A., Maguire, J., Birken, C., & Parkin, P. (2016, Oct-Dec). Diagnostic accuracy of developmental screening in primary care at the 18-month health supervision visit: A cross-sectional study. *CMAJ Open, 4*(4), E634-e640. https://doi.org/10.9778/cmajo.20160085

van Duijn, G., Dijkxhoorn, Y., Noens, I., Scholte, E., & van Berckelaer-Onnes, I. (2009). Vineland screener 0–12 years research version (nl). Constructing a screening instrument to assess adaptive behaviour. *Int J Methods Psychiatr Res, 18*(2), 110-117. https://doi.org/https://doi.org/10.1002/mpr.282

Van Duijn, G., Dijkxhoorn, Y., Scholte, E., & van Berckelaer-Onnes, I. (2010). The development of adaptive skills in young people with down syndrome. *J Intellect Disabil Res, 54*(11), 943-954. https://doi.org/https://doi.org/10.1111/j.1365-2788.2010.01316.x

Velikonja, T., Edbrooke-Childs, J., Calderon, A., Sleed, M., Brown, A., & Deighton, J. (2017). The psychometric properties of the Ages & Stages Questionnaires for ages 2-2.5: A systematic review. *Child Care Health Dev, 43*(1), 1-17. https://doi.org/10.1111/cch.12397

Venter, A., & Bham, A. (2003). The usefulness of commercially available 'culture fair' tests in the assessment of educational success in grade 1 black pupils in south africa—an explorative study. *J Child Adolesc Ment Health, 15*(1), 33-37. https://doi.org/https://doi.org/10.2989/17280580309486538

Voigt, R. G., Llorente, A. M., Jensen, C. L., Fraley, J. K., Barbaresi, W. J., & Heird, W. C. (2007). Comparison of the validity of direct pediatric developmental evaluation versus developmental screening by parent report. *Clin Pediatr*, *46*(6), 523-529. https://doi.org/10.1177/0009922806299100

Voress, J. K., Maddox, T., & Hammill, D. (2013). *Developmental Assessment Of Young Children (2nd ed)*. PRO-ED.

Wachtel, R. C., Shapiro, B. K., Palmer, F. B., Allen, M. C., & Capute, A. J. (1994, 1994/07/01). CAT/CLAMS: A tool for the pediatric evaluation of infants and young children with developmental delay. *Clin Pediatr*, *33*(7), 410-415. https://doi.org/10.1177/000992289403300706

Wakabayashi, T., Claxton, J., & Smith, E. V. (2019). Validation of a revised observation-based assessment tool for children birth through kindergarten: The COR Advantage. *J Psychoeduc Assess*, *37*(1), 69-90. https://doi.org/10.1177/0734282917732491

Wake, M., Gerner, B., & Gallagher, S. (2005). Does parents' evaluation of developmental status at school entry predict language, achievement, and quality of life 2 years later? *Ambul Pediatr*, *5*(3), 143-149. https://doi.org/https://doi.org/10.1367/A04-162R.1

Wang, L.-W., Wang, S.-T., & Huang, C.-C. (2005). Validity of the Clinical Adaptive Test (CAT)/Clinical Linguistic And Auditory Milestone Scale (CLAMS) as a screening instrument for very low birth weight infants in Taiwan. *J Dev Behav Pediatr*, *26*(6), 412-418. https://doi.org/https://doi.org/10.1097/00004703-200512000-00004

Wang, T. (2003). The Comprehensive Developmental Inventory for Infants and Toddlers–manual. *Taipei: Special Education Division, Ministry of Education*.

Wetherby, A. M., Allen, L., Cleary, J., Kublin, K., & Goldstein, H. (2002). Validity and reliability of the Communication and Symbolic Behavior Scales Developmental Profile with very young children. *J Speech Lang Hear Res, 45*(6), 1202-1218. https://doi.org/10.1044/1092-4388(2002/097)

Wetherby, A. M., & Prizant, B. (2002). *Communication and Symbolic Behavior Scales Developmental Profile-first normed edition*. Baltimore, MD: Paul H. Brookes.

Wiggins, L. D., Piazza, V., & Robins, D. L. (2014). Comparison of a broad-based screen versus disorder-specific screen in detecting young children with an autism spectrum disorder. *Autism, 18*(2), 76-84. https://doi.org/https://doi.org/10.1177/1362361312466962

Wijedasa, D. (2012). Developmental screening in context: Adaptation and standardization of the Denver Developmental Screening Test-II (DDST-II) for Sri Lankan children. *Child Care Health Dev*, *38*(6), 889-899. https://doi.org/10.1111/j.1365-2214.2011.01332.x

Winchell, B. (2011). *A critical examination of the technical adequacy of a curriculum-based assessment using rasch analyses* ProQuest Dissertations Publishing].

Wong, A. M. Y., Leung, C., Siu, E. K. L., & Lam, C. C. C. (2012). Validating the language domain subtest in a developmental assessment scale for preschool children. *Res Dev Disabil, 33*(5), 1633-1641. https://doi.org/10.1016/j.ridd.2012.03.002

Wong, H. S., Santhakumaran, S., Cowan, F. M., & Modi, N. (2016). Developmental assessments in preterm children: A meta-analysis. *Pediatrics, 138*(2), 1-12. https://doi.org/10.1542/peds.2016-0251

Wu, J. (1997). *Language, play and general development for chinese infants-toddlers: Using adapted assessments*. University of Colorado at Boulder].

Yao, S.-Y., Bull, R., Khng, K. H., & Rahim, A. (2018). Psychometric properties of the NEPSY-II affect recognition subtest in a preschool sample: A rasch modeling approach. *Clin Neuropsychol, 32*(1), 63-80. https://doi.org/https://doi.org/10.1080/13854046.2017.1343865

Yu, Y.-T., Hsieh, W.-S., Hsu, C.-H., Chen, L.-C., Lee, W.-T., Chiu, N.-C., Wu, Y.-C., & Jeng, S.-F. (2013). A psychometric study of the Bayley Scales of Infant and Toddler Development – 3rd edition for term and preterm Taiwanese infants. *Res Dev Disabil, 34*(11), 3875-3883. https://doi.org/10.1016/j.ridd.2013.07.006

Yue, A., Jiang, Q., Wang, B., Abbey, C., Medina, A., Shi, Y., & Rozelle, S. (2019b). Concurrent validity of the Ages and Stages Questionnaire and the Bayley Scales of Infant Development III in China. *PLoS One, 14*(9), 1-20. https://doi.org/10.1371/journal.pone.0221675

Yunilda, E., Gunardi, H., Medise, B. E., & Oswari, H. (2023). The Indonesian version of Ages and Stages Questionnaire III accuracy compared to Bayley Scales of Infant Development III. *Infant Child Dev*, *32*(1), e2387. https://doi.org/https://doi.org/10.1002/icd.2387

Zirakashvili, M., Gabunia, M., Tatishvili, N., Ediberidze, T., Lomidze, G., Chachava, T., & Hix-Small, H. (2018). Cultural adaptation and psychometric validation of the Ages and Stages Questionnaires for use in Georgia. *J Child Fam Stud*, *27*, 739-749. https://doi.org/https://doi.org/10.1007/s10826-017-0917-z

Zwi, K., Rungan, S., Woolfenden, S., Williams, K., & Woodland, L. (2016). Methods for a longitudinal cohort of refugee children in a regional community in Australia. *BMJ Open*, *6*(8), e011387. https://doi.org/10.1136/bmjopen-2016-011387
